# CNGCs in *Marchantia paleacea* uncouple arbuscular mycorrhizal symbiosis and rhizoid development

**DOI:** 10.1111/nph.71296

**Published:** 2026-06-04

**Authors:** Anson Ho Ching Lam, Aisling Cooke, Jake Richardson, Myriam Charpentier

**Affiliations:** ^1^ Cell and Developmental Biology Department John Innes Centre Norwich NR4 7UH UK

**Keywords:** anisotropic cell expansion, arbuscular mycorrhizal symbiosis, bryophytes, calcium signalling, CNGC, *Marchantia*

## Abstract

- In *Marchantia paleacea*, MpaDMI1‐dependent nuclear Ca^2+^ oscillations are essential for arbuscular mycorrhizal (AM) fungal colonisation, indicating that endosymbiosis‐mediated nuclear Ca^2+^ signalling is a conserved feature of land plant–AM symbiosis. Despite this conservation, DOES NOT MAKE INFECTION (DMI)1 regulatory properties have diverged between bryophytes and angiosperms, suggesting lineage‐specific adaptation and incomplete conservation of the Ca^2+^ oscillation machinery. In angiosperms, DMI1‐dependent Ca^2+^ release requires CYCLIC NUCLEOTIDE‐GATED CHANNELS (CNGC)15, but whether a comparable CNGC module operates in bryophytes, whose CNGC gene family is greatly reduced, has remained unknown.
- Here, we combined phylogenetic, genetic and cell biology approaches to investigate diverging land plant CNGCs function.
- Phylogenetic analyses across streptophytes reveal that CNGCs diversified into three ancient superclades before the terrestrialisation of plants. Functional analyses reveal that in *M. paleacea*, the combined activity of three MpaCNGCs spanning two superclades is required for endosymbiosis‐associated nuclear Ca^2+^ oscillations and AM fungal colonisation. Although two of these MpaCNGCs redundantly regulate rhizoid elongation, AM fungi activate Ca^2+^ signalling and penetrate ventral cells lacking rhizoid growth, indicating that tip‐growing cells are not strictly required for fungal entry in *M. paleacea*.
- Together, these findings link MpaCNGC function in rhizoid development and AM symbiosis to nutrient acquisition, supporting both soil exploration and AM fungal colonisation.

## Graphical abstract

Rhizoid growth and AM fungal infection are uncoupled.

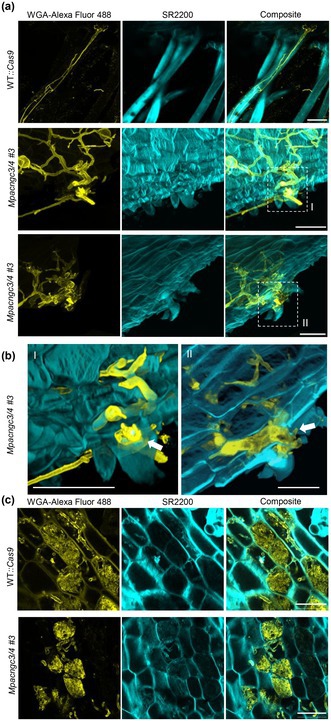

## Introduction

Arbuscular mycorrhizal fungi (AMF), belonging to the phylum Glomeromycota, constitute an ancient and widespread monophyletic fungal lineage that forms mutualistic endosymbiosis with the roots of the majority of terrestrial plants (Brundrett & Tedersoo, 2018; Rimington *et al*., 2018). In this association, AMF enhance host acquisition of water and essential mineral nutrients, such as phosphorus and nitrogen, in exchange for photosynthetically fixed carbon and lipids required for fungal growth and completion of their life cycle (Govindarajulu *et al*., 2005; Smith & Read, 2008; Keymer *et al*., 2017; Luginbuehl *et al*., 2017; Wang *et al*., 2017). Fossil evidence indicates that AM symbiosis originated > 400 million years ago (Ma) (Remy *et al*., 1994), suggesting that this partnership played a critical role in the colonisation of land by plants by facilitating their survival and expansion in nutrient‐poor terrestrial environments.

Among bryophytes, which represent nonvascular land plant lineages, colonisation by Glomeromycotan fungi is well documented, particularly in liverworts and hornworts while it is absent in mosses (Humphreys *et al*., 2010; Desiro *et al*., 2013; Rimington *et al*., 2018). Notably, the complex thalloid liverwort *Marchantia paleacea* exhibits well‐characterised AM associations, making it a valuable model for dissecting the cellular and genetic basis of plant–AM symbiosis (Bowman *et al*., 2022). In *M. paleacea*, AMF entry occurs through tip‐growing unicellular rhizoids before colonising the nonphotosynthetic storage region of the thallus, where highly branched arbuscules are formed (Ligrone *et al*., 2007).

Rhizoids share notable morphological and mechanistic similarities with the root hairs of vascular plants, despite their distinct developmental origins. Rhizoids arise from the gametophytic thallus, whereas root hairs develop from the sporophytic root (Jones & Dolan, 2012). Both cell types undergo polarised growth, contribute to anchorage, water and phosphate uptake, and rely on similar molecular components for their development (Menand *et al*., 2007; Honkanen *et al*., 2016; Kanno *et al*., 2026). In vascular plants, AMF penetration of root epidermal cells can occur independently of the presence of root hairs (Guinel & Hirsch, 2000). Fungal entry takes place in both trichoblast (root hair cell) and atrichoblast (nonroot hair cell), with the frequency of trichoblast infection varying among species (Guinel & Hirsch, 2000; Novero *et al*., 2008). In trichoblasts, penetration is mainly localised to the basal region of the cell rather than the tubular outgrowth itself (Novero *et al*., 2008). These observations raise the question of whether AMF entry in *M. paleacea* strictly requires a tubular rhizoid structure.

At the molecular level, colonisation of vascular plant roots by AMF is governed by the symbiotic nuclear calcium (Ca^2+^) signalling pathway (Bonfante & Genre, 2010). Perception of diffusible fungal signals, such as short‐chain chitooligosaccharides, by plasma membrane receptor‐like kinases triggers oscillations in nuclear Ca^2+^ concentration (Genre *et al*., 2013; Feng *et al*., 2019; He *et al*., 2019; Zhang *et al*., 2024; Tan *et al*., 2025; Teyssier *et al*., 2025). These Ca^2+^ oscillations initiate transcriptional reprogramming of host cells, thereby enabling the intracellular accommodation of the fungal endosymbiont. In the model legume *Medicago truncatula*, nuclear Ca^2+^ oscillations are generated by a multiprotein complex of nuclear envelope‐localised ion channels, including DOES NOT MAKE INFECTION (DMI1) and CYCLIC NUCLEOTIDE‐GATED CHANNELS 15a, 15b and 15c (CNGC15a/b/c), which together regulate Ca^2+^ efflux across the inner nuclear membrane (Ane *et al*., 2004; Charpentier *et al*., 2016; Cook *et al*., 2025). The oscillatory dynamics of nuclear Ca^2+^ release are jointly regulated by DMI1, which function as an activator of the CNGC15a/b/c‐mediated nuclear calcium release, and by CALMODULIN2, which acts as a Ca^2+^‐dependent negative regulator by promoting CNGC15a/b/c closure following each release event (Del Cerro *et al*., 2022; Cook *et al*., 2025).

In *M. paleacea*, MpaDMI1‐mediated nuclear Ca^2+^ oscillations are likewise required for successful AM fungal colonisation of the thallus but are dispensable for fungal hyphal penetration of rhizoids (Lam *et al*., 2024). This demonstrates that nuclear Ca^2+^ oscillation represents a conserved component of land plant–AM symbiosis, whereas rhizoid infection of bryophytes occurs via a nuclear Ca^2+^ oscillation‐independent pathway. Despite this functional conservation in thallus colonisation, the gating properties of DMI1 have diverged between angiosperms and bryophytes (Lam *et al*., 2024), indicating that while the core role of DMI1 in coordinating symbiotic Ca^2+^ oscillations has been maintained, the molecular mechanism underlying channel regulation has undergone lineage‐specific adaptation. This divergence further suggests that the molecular architecture of the Ca^2+^ oscillation machinery may not be fully conserved. In *M. truncatula*, CNGC15 channels function as key effectors of DMI1‐dependent Ca^2+^ release, yet whether an equivalent CNGC‐mediated module operates in bryophytes remains unknown. Notably, the CNGC gene family is markedly reduced in bryophytes compared with flowering plants (Wheeler & Brownlee, 2008), in which CNGCs participate in diverse signalling pathways, including development and responses to biotic and abiotic stresses (DeFalco *et al*., 2016; Tan *et al*., 2020; Tipper *et al*., 2023; Ming *et al*., 2025). Together, these observations raise the question of whether CNGCs originally evolved to support symbiotic Ca^2+^ signalling and AM fungal colonisation, or whether they served alternative functions and were subsequently recruited in angiosperms to support endosymbiosis. To address this, we investigated the role of CNGCs in *M. paleacea* during AM symbiosis through an integrated phylogenetic, genetic and cell biology analysis.

## Materials and Methods

### Phylogenetic analysis

**Fig. 1:**
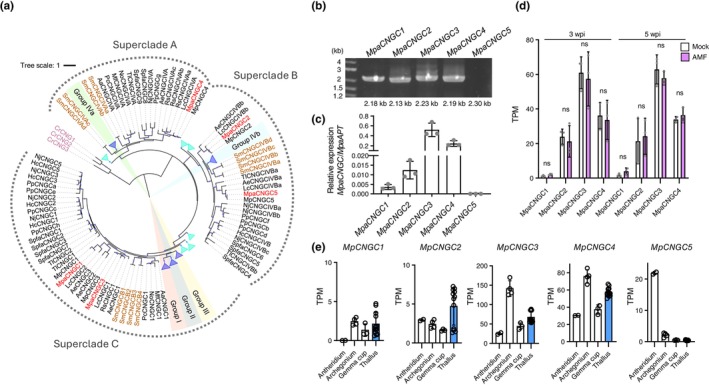
MpaCNGC3 and MpaCNGC1 cluster within superclade C, including *Medicago truncatula* CYCLIC NUCLEOTIDE‐GATED CHANNELS (CNGC)15s. (a) Maximum‐likelihood phylogeny of CNGC amino acid sequences from 11 angiosperm species collapsed into Groups I, II, III, IVa and IVb (shown in blue triangles); one lycophyte (Sm, *Selaginella moellendorffii*); 14 bryophytes (Aa, *Anthoceros agrestis*; Ae, *Apopellia endiviifolia*; Hc, *Hypnum curvifolium*; Lc, *Lunularia cruciata*; Mf, *Megaceros flagellaris*; Mp, *Marchantia polymorpha*; Mpa, *Marchantia paleacea*; Nj, *Niphotrichum japonicum*; No, *Notothylas orbicularis*; Pc, *Phaeoceros carolinianus*; Pp, *Physcomitrium patens*; Rs, *Riccia sorocarpa* Bisch.; Spfa, *Sphagnum fallax*; Tl, *Takakia lepidozioides*); 26 charophytes collapsed and shown in cyan triangles. The tree is rooted using CNGCs from a chlorophyte (Cr., *Chlamydomonas reinhardtii*), labelled in pink. *Medicago paleacea* CNGCs are highlighted in red, and lycophyte CNGCs in brown. The amino acid sequences are listed in Supporting Information Table S1, and the uncollapsed phylogeny is presented in Fig. S1. Blue dots on branches represent ultrafast bootstrap support values > 90% (1000 iterations). The bar indicates substitutions per site. (b) Reverse transcription polymerase chain reaction analysis showing the absence of detectable full‐length *MpaCNGC5* transcripts in 3‐wk‐old *M. paleacea* thalli. The expected sizes of full‐length *MpaCNGCs* are indicated below. (c) Scatter dot plot with bars representing real‐time quantitative polymerase chain reaction analysis of *MpaCNGC* transcript levels in 3‐wk‐old *M. paleacea* plants. Each point represents a biological replicate with cDNA produced from at least 20 plants. The housekeeping gene *ADENINE PHOSPHORIBOSYL TRANSFERASE* (*APT*) was used for normalisation. Bars and error bars indicate the mean and SD, respectively. (d) Scatter dot plot with bars representing expression analysis of *MpaCNGCs* based on RNA‐seq data generated at 3 and 5 wk postinoculation (wpi) with *Rhizophagus irregularis* from Rich *et al*. (2021). The transcript levels are presented as transcripts per million (TPM). Bars and error bars indicate the mean and the SD, respectively. Statistical significance was assessed using a Wald test implemented in DESeq2, comparing mock and arbuscular mycorrhizal fungi (AMF) treatment. ns, nonsignificant. (e) Scatter dot plot with bar representing the expression of *M. polymorpha CNGCs*, shown as TPM, across 21‐d‐old *M. polymorpha* thalli and gemma cup, and 13‐d‐old reproductive organs (antheridium and archegonium). Bars and error bars indicate the mean and the SD, respectively. Gene expression data were retrieved from http://bar.utoronto.ca/efp_marchantia/cgi‐bin/efpWeb.cgi.

CNGC sequences were identified through BLASTp searches against genomes from 1KP project (Carpenter *et al*., 2019; Leebens‐Mack James *et al*., 2019), Phytozome (Goodstein *et al*., 2012) and genomes from *Marchantia* (Montgomery *et al*., 2020; Rich *et al*., 2021), *Apopellia endiviifolia* Dicks. (Szablinska‐Piernik *et al*., 2026), *Lunularia cruciate* (Linde *et al*., 2023), *Riccia sorocarpa* Bisch. (Krawczyk *et al*., 2025), hornworts (Schafran *et al*., 2025), *Hypnum curvifolium* Hedw. (Yu *et al*., 2022), *Niphotrichum japonicum* Dozy & Molk. (Zhou *et al*., 2023) and *Takakia lepidozioides* S.Hatt. & Inoue (Hu *et al*., 2023). The *M. truncatula* CNGC sequences were used as input for Blastp. Blast+ v(2.7.1) was used for genomes without a web server (Camacho *et al*., 2009), followed by reciprocal Blastp. Accessions and amino acid sequences included in the analysis are listed in Supporting Information Table S1. Multiple sequence alignment was performed with Mafft with the L‐INS‐I algorithm (Katoh & Standley, 2013). Maximum‐likelihood phylogenetic analyses were constructed using IQ‐Tree v.2.3.2 (Nguyen *et al*., 2015). The best‐fitting substitution model was selected using ModelFinder (Kalyaanamoorthy *et al*., 2017), and branch support was assessed using 1000 ultrafast bootstrap replicates (UFBoot2; Hoang *et al*., 2018). The JTT + F + R7 model was used for tree reconstruction. Trees were visualised using the interactive Tree Of Life (iTOL) v.6 (Letunic & Bork, 2024). Collapsed clades in Fig. 1(a) are displayed as triangles.

### *MpCNGC* expression analysis

For reverse transcription polymerase chain reaction (RT‐PCR) analysis, total RNA was extracted from 3‐wk‐old *Marchantia paleacea* thalli using RNeasy Plant Mini Kit (Qiagen) according to the manufacturer's instructions. Genomic DNA was digested using Ambion Turbo DNA‐free Kit (Thermo Scientific, Waltham, MA, USA) according to the manufacturer's instructions. cDNA was synthesised from RNA using SuperScript IV Reverse Transcriptase (Invitrogen) according to the manufacturer's instructions with 4 U μl^−1^ reverse transcriptase used per 20 μl reaction. PCR amplification was performed using Phusion DNA polymerase (Thermo Scientific) and primers spanning the full‐length coding region of *MpaCNGCs* (Table S2).

For real‐time quantitative polymerase chain reaction (RT‐qPCR) analysis, total RNA was isolated from 3‐wk‐old *Marchantia paleacea* plants, and cDNA was synthesised as above. Quantitative PCR was performed using gene‐specific primers for *MpaCNGCs* listed in Table S2. Transcript abundance was normalised to the housekeeping gene *ADENINE PHOSPHORIBOSYL TRANSFERASE* (*MpaAPT*). Relative expression levels were calculated using the $2^{-△△C_{T}}$ method according to (Schmittgen & Livak, 2008).

RNA‐seq‐based expression analysis of *MpaCNGCs* during AM symbiosis was performed using publicly available datasets generated at 3 and 5 wk post inoculation (wpi) with *Rhizophagus irregularis* (Rich *et al*., 2021). Reads were mapped to the *Marchantia paleacea* genome using Hisat2 v.2.1.0 (Kim *et al*., 2019). SAMtools v.1.4.1 was used to process and index alignment files (Li *et al*., 2009). Stringtie v.1.3.5 was used to count gene counts (Pertea *et al*., 2015). DESeq2 v.1.42.1 was used for testing differential gene expression (Love *et al*., 2014).

Expression profiles of *M. polymorpha CNGCs* across different developmental stages and tissues were obtained from the *Marchantia* eFP Browser (http://bar.utoronto.ca/efp_marchantia/cgi‐bin/efpWeb.cgi) (Tan *et al*., 2023). Transcripts per million (TPM)‐normalised expression values were extracted for 21‐d‐old thalli and gemma cups, as well as 13‐d‐old reproductive organs (antheridia and archegonia).

To examine the spatial expression patterns of *MpaCNGC*s, promoter regions upstream of the translation start site were synthesised by GenArt (Life Technologies) and fused to the *β‐GLUCURONIDASE* (*GUS*) reporter gene using Golden Gate cloning strategy (Table S3; Engler *et al*., 2009; Weber *et al*., 2011). BsaI‐HFv2 (New England Biolabs, Ipswich, MA, USA) or Eco31I (Thermo Scientific) was used for making Level 1 plasmids. BpiI (Thermo Scientific) was used for making Level 2 plasmids. Promoter fragments of 1.5 kb (*MpaCNGC1*), 1.5 kb (*MpaCNGC2*), 2.0 kb (*MpaCNGC3*) and 1.5 kb (*MpaCNGC4*) were synthesised by GenArt (Life Technologies). Accession numbers of *MpaCNGCs* are listed in Table S4. The resulting promoter‐GUS constructs, inserted in Position 2 and flanked by *HYGROMYCIN PHOSPHOTRANSFERASE* in Position 1 and *mcherry* in Position 3 (Table S3) were introduced into *M. paleacea* using transformation procedures as described previously (Lam *et al*., 2024). Transgenic plants were selected based on their ability to grow on half‐strength Gamborg's B5 supplemented with 20 μM hygromycin B (Thermo Fisher, Waltham, MA, USA) and to express mCherry fluorescence. The transgenic plants were grown under conditions described below, and GUS activity was analysed in 2‐wk‐old thalli as described previously (Lam *et al*., 2024). Samples were imaged using either a Leica DM6000 or Leica M205FA Stereo microscope.

### Plant materials and growth conditions

*Marchantia paleacea* plants were grown in Percival Scientific growth chamber (model CU‐36 L4) under a long‐day photoperiod (16 h, 22°C/8 h, 18°C) with white light at an intensity of 60 mmol m^−2^ s^−1^. For propagation, plants were cultured on half‐strength Gamborg's B5 medium supplemented with MgSO_4_, solidified with 0.8% (w/v) agar, and without sucrose. The medium contained 12.4 mM KNO_3_, 3 mM MgSO_4_·, 0.51 mM CaCl_2_, 0.51 mM (NH_4_)_2_SO_4_, 58.6 μM NaFe‐EDTA, 29.6 μM MnSO_4_, 544 μM NaH_2_PO_4_, 24.3 μM H_3_BO_3_, 3.48 μM ZnSO_4_, 2.26 μM KI, 517 nM Na_2_MoO_4_·, 50 nM CuSO_4_·, 278 μM Myo‐inositol, 14.8 μM Thiamine, 2.43 μM Pyridoxine and 4.06 μM Nicotinic acid.

### Generation of *M. paleacea* CRISPR/Cas9 mutants

Clustered Regularly Interspaced Short Palindromic Repeats (CRISPR)/CRISPR‐associated protein 9 (Cas9) constructs used in this study were generated using Golden Gate cloning and are listed in Table S3.

Guide RNA (sgRNA) target sites were selected using sgRNA Scorer 2.0 (Chari *et al*., 2017) based on genomic sequences of the target genes. The selected gRNA sequences are highly specific, such that any potential off‐target sites in the genome differ by more than three nucleotides from the intended target and are therefore unlikely to be targeted. Single‐stranded DNA containing gRNA sequences was synthesised by Integrated DNA Technologies (Coralville, IA, USA). Annealed double‐stranded oligonucleotides encoding gRNAs were cloned downstream of the *MpaU6‐1* promoter using Golden Gate assembly using Esp3I/BsmBI and BsaI to generate Level 1 *pMpaU6‐1:gRNA:tracrRNA* vectors, which were subsequently assembled into Level 2 CRISPR/Cas9 constructs (Table S3). Cloning of the promoter of *MpaU6‐1* and the chlorsulphuron resistance gene *ACETOLACTATE SYNTHASE* (*mALS*) was described in Lam *et al*. (2024).

Stable transformation of *M. paleacea* was performed using *Agrobacterium tumefaciens* strain AGL1, following the protocol described in Lam *et al*. (2024).

The selection of the *M. paleacea* CRISPR/Cas9 mutants was performed as followed. Antibiotic‐resistant transgenic plants expressing nuclear‐localised Yellow Cameleon 3.6 (NLS:YC3.6) were screened for fluorescence using a Leica DMR/MZFLIII microscope. Genomic DNA was extracted, and target loci were amplified by PCR as described previously (Lam *et al*., 2024). Primers used for genotyping are listed in Table S2. PCR products were subjected to Sanger sequencing (Azenta Life Sciences, Burlington, MA, USA), and sequence alignments were performed using Benchling (Biological software: https://benchling.com).

### Rhizoid length and thalli surface area measurement

For rhizoid length measurement, gemmae or 3 × 3‐mm thallus pieces, each containing a meristem, were grown on SRV media (Fonseca *et al*., 2006) solidified with 1% (w/v) agar in 90‐mm Petri dishes positioned vertically for 20 or 14 d, respectively. Plant Images were captured using a Zeiss Axio Zoom V16 microscope (Carl Zeiss, Cambourne, UK). The longest rhizoids were measured using ImageJ plugin NeuronJ (Meijering *et al*., 2004; Schindelin *et al*., 2012).

For thalli surface area measurement, cut thalli fragments were grown on each 90‐mm Petri dish containing 30‐ml half‐strength Gamborg's B5 + MgSO4 0.8% (w/v) agar and without sucrose media for 4 wk. The plants were imaged with a Zeiss Axio Zoom V16 microscope, and the surface area was calculated using ImageJ using the threshold tool (Schindelin *et al*., 2012).

### Cryo‐scanning electron microscopy

*Marchantia paleacea* thalli were mounted on an aluminium stub with Tissue Tek OCT (Calibre Scientific, Rotherham, UK) and plunge frozen in slushed liquid nitrogen to cryo‐preserve the material before transfer to the cryo‐stage of an ALTO 2500 cryo‐transfer system (Gatan, Oxford, UK) attached to an FEI Nova NanoSEM 450 (FEI, Eindhoven, the Netherlands). Surface frost was sublimated by warming the sample to −95°C for 4 min, before the sample was cooled to −125°C and sputter coated with platinum for 150 s at 10 mA. The sample was loaded onto the cryo‐stage of the main SEM chamber and held at −125°C during imaging at 3 kV using an Everhart–Thornley detector.

### Arbuscular mycorrhizal fungi colonisation

Plants were grown in 1‐l vented containers (DELItainer; Pactiv Evergreen, Lake Forest, IL, USA) containing 200 ml of inoculated soil mixture composed of 20% chive AMF inoculum mixed with sterile Terragreen 40% and sand 40% (Oil‐Dri Co., Chicago, IL, USA), with 10 plants per container. After 8 wk, *M. paleacea* thalli were embedded in 6% agarose and sectioned into 100–200 μm slices using a VT1000 vibratome (Leica Microsystems, Milton Keynes, UK). Sections were cleared in 10% (w/v) KOH overnight at 30°C, washed three times with water and stained in a solution of 5% (v/v) acetic acid and 3% (v/v) blue ink (Waterman, France) for 10–20 min. The sections were destained in water. Sections were imaged using a Zeiss Axio Imager Z2 microscope equipped with 10× or 20× air objectives.

For quantifying colonisation levels, at 8 wpi, *M. paleacea* thalli were harvested, cleaned and rhizoids attached to soil debris were removed. RNA extraction, cDNA synthesis and quantitative real time PCR (qRT‐PCR) were performed as described above. Transcript abundance was normalised to the housekeeping genes *ADENINE PHOSPHORIBOSYL TRANSFERASE* (*MpaAPT*) and *ELONGATION FACTOR 1α* (*MpaEF1α*). Relative expression levels were calculated using the equation: $E_{\text{target}}^{\left(-{\mathrm{Ct}}_{\text{target}}\right)}/\surd \left(E_{\mathrm{APT}}^{\left(-{\mathrm{Ct}}_{\mathrm{APT}}\right)}*E_{\mathrm{EF}1\alpha }^{\left(-{\mathrm{Ct}}_{\mathrm{EF}1\alpha }\right)}\right)$. *E* denotes the PCR efficiency. Primers used are listed in Table S2. Accession numbers of *MpaAPT*, *MpaEF1α*, *MpaSTR*, *MpaAMT2*, *MpaSCR* and *RiEF1a* are listed in Table S4.

For imaging of infection events in WT::*Cas9* and *Mpacngc3/4* mutants, the plants were sectioned, cleared and stained with ink as above. The sections with AM fungal infections were then stained with 0.5 μg ml^−1^ WGA‐Alexa Fluor 488 and 0.2% Renaissance SR2200 in 1× PBS overnight at room temperature.

For percentage AMF thalli length colonisation, *M. paleacea* thalli were cleared in 10% (w/v) KOH for 5 d at 37°C and then washed with water. The thalli were bleached in 7.5% (v/v) hydrogen peroxide (pH 9) for 8 h, followed by two washes with water. They were then stained with 0.01% aniline blue in 95% acetic acid for 24 h, washed with water and destained in 80% acetic acid. Subsequently, the thalli were washed with phosphate‐buffered saline and stained overnight at room temperature in the dark with 0.5 μg ml^−1^ WGA‐Alexa Fluor 488 (Thermo Scientific). Imaging was performed using a Zeiss Axio Zoom V16 microscope (Carl Zeiss) equipped with a 38 HE GFP filter (excitation: 450–490 nm; emission: 500–550 nm). Quantification of AMF colonisation within each thallus was performed using ImageJ. The extent of AMF colonisation, visualised via WGA‐Alexa Fluor 488 staining, was measured as the length of colonised regions and expressed relative to the total thallus length, defined as the distance from the basal region to the apical notches.

### Confocal laser scanning microscopy

Fluorescence imaging was performed using a Zeiss LSM980 confocal laser scanning microscope equipped with a 25×/0.8 multi‐immersion objective with water immersion. WGA‐Alexa Fluor 488 was excited at 514 nm, and Renaissance SR2200 was excited at 405 nm. Sequential scanning was used and emission was captured at 380–608 nm with Airyscan 2 detectors. Three‐dimensional visualisation was performed with the Zeiss Arivis Vision4D software.

For simultaneous imaging of YC3.6 and Renaissance SR2200, cpYFP was excited at 514 nm and Renaissance SR2200 at 405 nm. Sequential scanning was used and emission was captured at 420–480 and 495–550 nm with Airyscan 2 detectors.

### Generation of germinating spore exudate

Six thousand sterile spores of *Rhizophagus irregularis* (PTB297‐L‐ASP‐A; Premier Tech, Riviere‐du‐Loup, QC, Canada) were incubated in 30 ml water at 30°C for 2 wk in the dark. After spore germination, the supernatant was collected, concentrated 50‐fold using a Virtis lyophilizer, aliquoted and stored at −20°C.

### Calcium imaging

Nuclear calcium oscillations were measured using a Nikon ECLIPSE FN1 microscope equipped with an emission image splitter (OptoSplit II; Cairn Research, Faversham, Kent, UK) and an electron‐multiplying cooled charge‐coupled device (EMCDD) camera (Rolera Thunder; QImaging, Calgary, AB, Canada). ECFP was excited using light emitting diode (OptoLED; Cairn research) at 436 ± 20 nm and emitted fluorescence detected at 535 ± 30 nm (cpVenus) and 480 ± 40 nm (ECFP). Images were acquired at 3‐s intervals for at least 1 h using the MetaFluor software. Two‐ to three‐week‐old *M. paleacea* gemmae grown vertically on SRV solidified with 1% (w/v) agar were mounted in a chamber assembled on a 76 × 26 mm coverglass (Solmedia, Shrewsbury, Shropshire, UK) using high‐vacuum grease (Dow Corning GMBH, Wiesbaden, Germany). Approximately 80 μl of liquid SRV medium was added to the chamber, and samples were incubated for 30 min before imaging. After 10–20 min of baseline imaging, 10 μl of 50× concentrated AM fungal germinated spore exudate was added.

### Statistical analysis

Statistical significance was assessed by Kruskal–Wallis test with multiple comparisons against WT::*Cas9* as indicated in figure legends. Statistical tests were performed using GraphPad Prism v.8.

## Results

### MpaCNGC1/3 are co‐orthologues of MtCNGC15a/b/c

*Marchantia paleacea* genome encodes five *CNGC* genes, matching the *CNGC* copy number reported for the non‐AMF host *M. polymorpha* (Marchetti *et al*., 2021). By contrast, angiosperm genomes show substantial expansion of this gene family; *Arabidopsis thaliana* and *M. truncatula* possess 20 and 23 *CNGCs*, respectively (Charpentier *et al*., 2016). To identify the orthologue of *MtCNGC15s* in *M. paleacea*, we performed a maximum‐likelihood phylogenetic analysis based on CNGC amino acid sequences from streptophytes, including 26 species of land plants and 26 species of charophytes. The phylogeny was rooted with CNGCs from the chlorophyte *Chlamydomonas reinhardtii*. Previous phylogenetic analyses, based largely on angiosperms, resolved CNGCs into five lineage‐specific groups (Groups I‐IVb; Maser *et al*., 2001), shaped by gene expansion and duplication within flowering plants (Fig. S1). By contrast, the inclusion of charophyte, bryophyte and lycophyte representatives in our analysis revealed that land plant CNGCs cluster into three ancient superclades, designated A to C (Figs 1a, S1). This topology including the charophytes demonstrates that the major diversification of the CNGC family predates the emergence of land plants, and consequently, of AM symbiosis. Thus, the apparent group‐level complexity observed in angiosperms largely reflects secondary expansion and specialisation of a small number of CNGC lineages inherited from algal ancestors.

The superclade C comprises Groups I, II and III, whereas Groups IVa and IVb fall into the distinct superclades A and B, respectively (Figs 1a, S1). The five *M. paleacea* CNGCs are distributed across these superclades: MpaCNGC1 and MpaCNGC3 branch within the superclade C, that includes Group III CNGC15a, CNGC15b and CNGC15c paralogs, while MpaCNGC4 belongs to superclade A, and MpaCNGC5 and MpaCNGC2 cluster within superclade B. This phylogenetic distribution of MpaCNGCs suggests that these channels might retain functional properties that predate the extensive diversification observed in angiosperms. The placement of MpaCNGC1 and MpaCNGC3 within the superclade C further indicates that they are the closest orthologues of CNGC15a, b, c. Altogether, the phylogenetic analysis reveals that the CNGC family in *M. paleacea* comprises a limited, yet an evolutionary representative subset of CNGCs that predate land plant diversification. However, unlike angiosperms, in which gene expansion and duplication have enabled functional specialisation and subfunctionalisation, the limited CNGC repertoire in bryophytes suggest that individual channels may fulfil broader or multifunctional roles, potentially integrating symbiotic Ca^2+^ signalling with other cellular processes. The close phylogenetic relationship between MpaCNGC1 and MpaCNGC3 and the angiosperm CNGC15a/b/c further raises the possibility that these channels may contribute to symbiotic nuclear Ca^2+^ oscillations in *M. paleacea*.

### MpaCNGC1, 3 and 4 are expressed in tissues susceptible to AMF colonisation

To determine whether *MpaCNGC1* and *MpaCNGC3* are expressed in tissues colonised by AMF, we first assessed their expression in 3‐wk‐old *M. paleacea* thalli. Among the five *M. paleacea CNGCs*, full‐length transcripts were detected for only four, namely *MpaCNGC1*, *MpaCNGC2*, *MpaCNGC3* and *MpaCNGC4* (Fig. 1b,c). Analysis of publicly available transcriptomic datasets (Rich *et al*., 2021) from 3‐ and 5‐wk‐old *M. paleacea* thalli grown in absence or presence of AMF demonstrated that the expression levels of each *CNGC* detected in thallus were not influenced by AM fungal colonisation (Fig. 1d). Spatial expression analysis using endogenous promoter‐*GUS* reporter lines further revealed that *pMpaCNGC1*, *pMpaCNGC3* and *pMpaCNGC4* drive expression of *GUS* in both the nonphotosynthetic storage region of the thallus (the midrib) and rhizoids (Fig. S2). By contrast, *pMpaCNGC2* driving *GUS* expression showed GUS activity in rhizoids, and expression was largely confined to discrete unknown cell types but absent from the midrib in 2‐wk‐old thalli (Fig. S2). Consistent with this finding, analysis of *M. polymorpha* gene expression atlas confirmed that *MpCNGC1*, *MpCNGC2*, *MpCNGC3* and *MpCNGC4* are expressed in thallus, and indicates that *MpCNGC5* expression is restricted to reproductive structures during *Marchantia polymorpha's* life cycle (Kawamura *et al*., 2022; Fig. 1e). Together, these expression data indicate that *MpaCNGC1*, *MpaCNGC3* and *MpaCNGC4*, but not *MpaCNGC5* and *MpaCNGC2*, are expressed in tissues relevant to AM fungal colonisation, the midrib and rhizoid.

### *Mpacngc3/4* double mutant is impaired in rhizoid and thallus development

Phylogenetic analysis indicates that both MpaCNGC1 and MpaCNGC3 are the closest orthologues of MtCNGC15s. This suggests that, despite the reduced number of CNGCs in *Marchantia*, more than one CNGC may function in AM symbiosis, provided that this function is conserved. Conversely, the limited size of the CNGC family also implies that individual MpaCNGCs may have retained additional functions. Notably, angiosperm CNGCs belonging to superclade C are implicated in root hair growth (Brost *et al*., 2019; Tan *et al*., 2020) and primary root development (Tipper *et al*., 2023), whereas members of superclade A are involved in plant architecture (Tan *et al*., 2024), suggesting that CNGCs may also contribute to the regulation of developmental processes in liverworts.

**Fig. 2:**
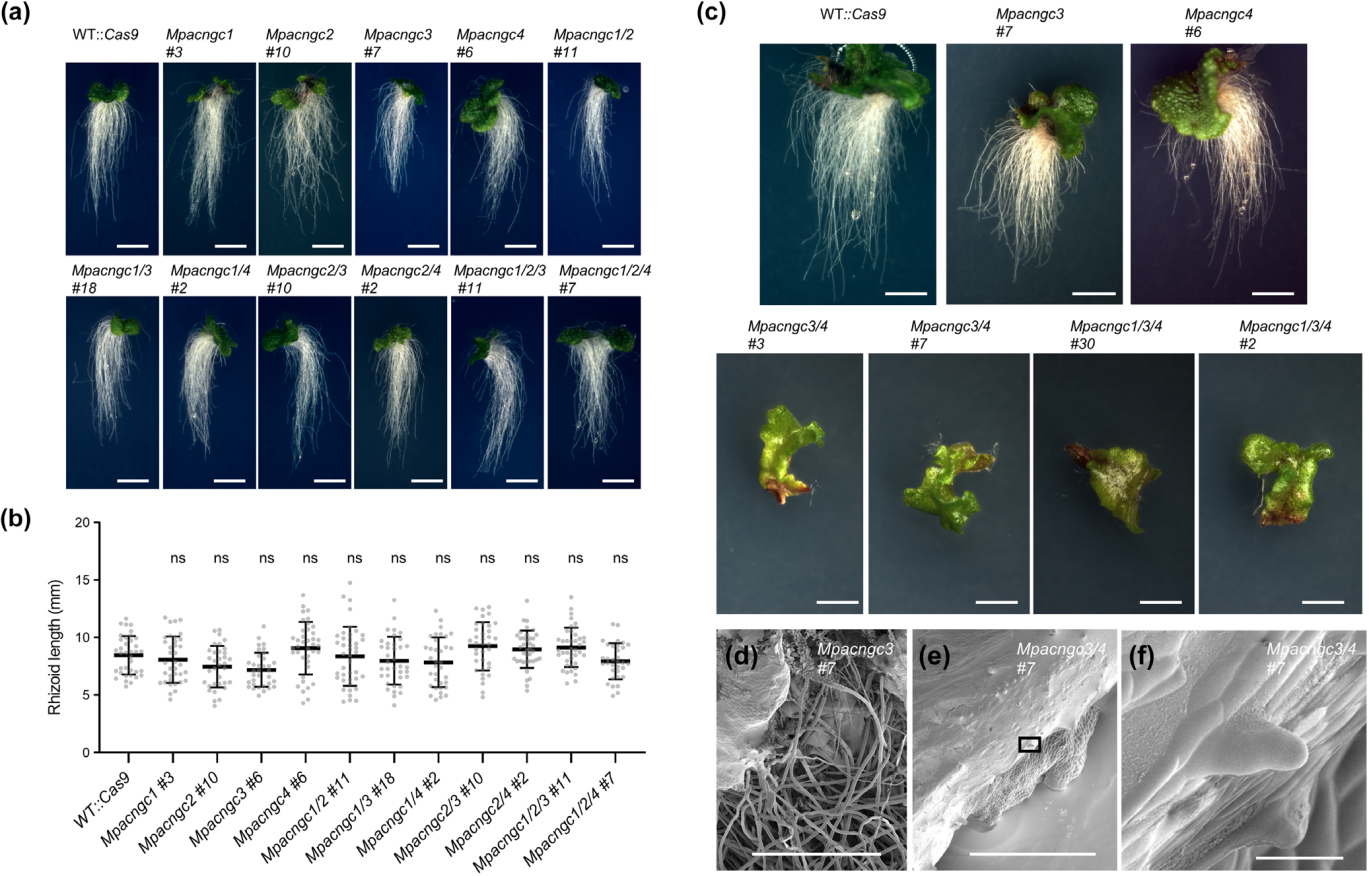
*Mpacngc3/4* mutants are defective in rhizoid development. (a) Images of 20‐d‐old *Medicago paleacea* wild‐type (WT) expressing *Cas9* (WT::*Cas9*) and *Mpacngc* mutants grown from gemmae on vertical plates. Bars, 2 mm. (b) Scatter dot plot showing the rhizoid lengths of 20‐d‐old *M. paleacea* WT::*Cas9* and *Mpacngc* mutants grown from gemmae. Bars and error bars indicate the mean and the SD, respectively. Statistical significance was assessed using the Kruskal–Wallis test with multiple comparisons against WT::*Cas9*. *n* ≥ 33. ns, not significant. Data from three biological replicates are shown. (c) Images of 2‐wk‐old *M. paleacea* WT::*Cas9* and *Mpacngc* mutants regenerated from cut thalli and grown on vertical plates. Bars, 2 mm. (d–f) Scanning electron microscope images of lower epidermis of thalli from (d) *Mpacngc3 #7* and (e, f) *Mpacngc3/4 #7* mutant. (f) Higher‐magnification image of the boxed region in (e). Bars, 1 mm in (d, e) and 50 μm in (f).

To assess their potential impact on the development of rhizoid and thallus, that are essential for AM fungal colonisation, we generated single and higher‐order *Mpacngc* mutants using a CRISPR/Cas9‐based strategy (Fig. S3). All single, double, triple and quadruple mutants obtained were confirmed by sequencing to be null alleles for their respective targeted *CNGC* genes (Figs S3, S4). Measurement of the rhizoid length from 20‐d‐old thalli grown vertically showed that the loss of individual *MpaCNGC* had no impact on rhizoid growth (Fig. 2a,b). However, higher‐order mutants exhibited much stronger phenotypes. In particular, the combined knockout of *MpaCNGC3* and *MpaCNGC4* resulted in defects in both rhizoid and thallus development. As *Mpacngc3/4* mutants failed to produce gemma cups, thallus surface area was quantified using cutting‐propagated thalli from independent mutant lines. After 4 wk of growth, *Mpacngc3/4* mutant thalli were half the size of wild‐type (WT) and single mutant thalli (Fig. S5a,b), and no rhizoid growth was observed (Fig. 2c). In addition, the thalli growth defect of *Mpacngc3/4* was not exacerbated in the triple mutants (*Mpacngc1/3/4* and *Mpacngc*2/3/4) or in the quadruple mutant *Mpacngc1/2/3/4* (Fig. S5), demonstrating that MpaCNGC3 and MpaCNGC4 modulate thallus development with no additional contribution from MpaCNGC1 and MpaCNGC2. Scanning electron microscopy analysis further revealed that rhizoid cells in *Mpacngc3/4* mutants were reduced to small bulges of *c*. 50 μm in length, indicating an essential and redundant role for MpaCNGC3 and MpaCNGC4 in rhizoid cell elongation. Altogether, our findings demonstrate that MpaCNGC3 and MpaCNGC4 play essential roles in both rhizoid and thallus development in *M. paleacea*. Although impaired rhizoid function could indirectly affect thallus growth via reduced water and nutrient uptake, a previous study identified transcription factors that impair rhizoid development without affecting thallus growth (Proust *et al*., 2016), suggesting that the observed thallus defects are unlikely to be solely secondary. Together, these results reveal that CNGCs possess developmental functions before the emergence of flowering plants. Furthermore, our findings highlight functional conservation between angiosperms and bryophytes despite *c*. 400 Ma divergence, with CNGC being required for the outgrowth of both root hair and rhizoid cells.

### *Mpacngc3/4* double mutant uncouples rhizoid development and AM fungal colonisation

**Fig. 3:**
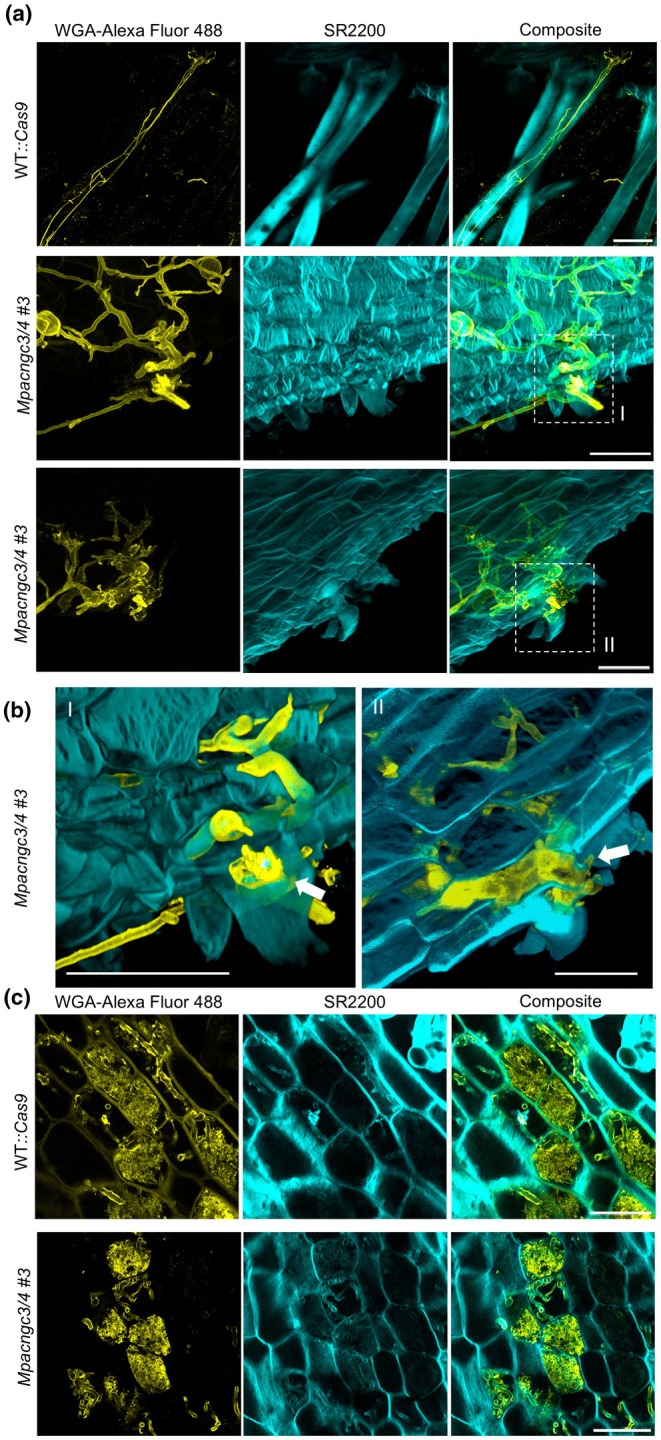
Rhizoid growth and arbuscular mycorrhizal (AM) fungal infection are uncoupled. (a–c) Confocal laser scanning microscopy images of cleared and stained sections of *Marchantia paleacea* WT::*Cas9* and *Mpacngc3/4 #3* plants colonised by *Rhizophagus irregularis* 6 wk postinoculation (wpi). The cyan channel shows the cell wall stain SR2200, and the yellow channel shows the AM fungi labelled with WGA‐Alexa Fluor 488. Bars, 50 μm (a) Maximum‐intensity projections of Z‐stacks showing AM fungal hyphae. (b) Zoomed‐in 3D‐cross section views of AM fungal infection events in *Mpacngc3/4 #3* mutant shown in (a), including (I) infection via bulged cell and (II) infection via nonbulged cell. White arrows indicate infection sites. (c) Representative images showing arbuscules developed within the thallus midrib of WT::*Cas9* and *Mpacngc3/4 #3* plants. WT, wild‐type.

AMF colonise *M. paleacea* via rhizoids (Kobae *et al*., 2019). To assess whether rhizoids are essential for AM fungal colonisation, we analysed infection in *Mpacngc3/4* mutants inoculated with *R. irregularis* for 6 wk. Confocal microscopy of WT::*Cas9* thallus sections co‐stained with the cell wall dye SR2200 and the fungal marker WGA‐Alexa Fluor 488 confirmed hyphal entry through rhizoids, followed by colonisation of the thallus midrib and formation of arbuscules (Fig. 3a,c). Despite the absence of developed rhizoids, AM fungal entry still occurred in *Mpacncg3/4* mutants via bulged and nonbulged cells, allowing hyphae to reach the thallus midrib and form arbuscules (Fig. 3a–c; Movies S1, S2). Collectively, these data demonstrate that, while rhizoids represent a major route for AM fungal entry in *M. paleacea*, they are not strictly required for successful colonisation.

### MpaCNGC1/3/4 are required for AM symbiosis

Endosymbiosis‐mediated nuclear Ca^2+^ oscillations are conserved across land plants and require the cation channel DMI1. Despite this functional conservation, the regulatory properties of DMI1 have diverged between bryophyte and angiosperms (Lam *et al*., 2024), raising the possibility that the molecular components underlying calcium oscillations are not fully conserved across land plant lineages.

**Fig. 4:**
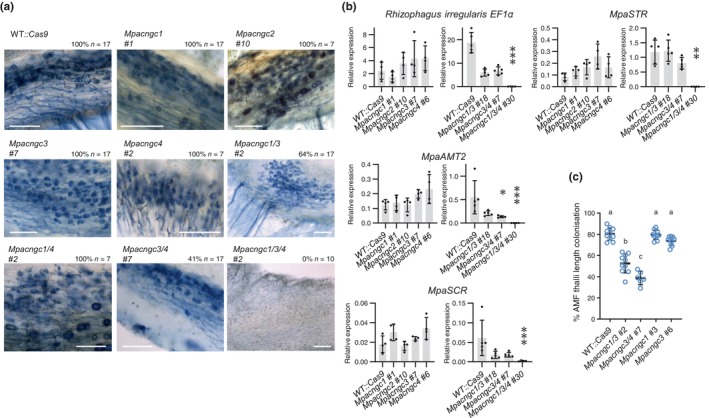
*Mpacngc1/3/4* mutants are impaired in arbuscular mycorrhizal (AM) symbiosis. (a) Representative pictures of transverse sections of *Medicago paleacea* thalli from wild‐type (WT)::*Cas9* and *Mpacngc* mutants 8 wk after inoculation with *Rhizophagus irregularis*. AM fungal structures were stained with acidic ink. The percentage of plants colonised is indicated with n denoting the total number of thalli analysed. The experiment was performed in three biological replicates. Bars, 200 μm. (b) Scatter dot plots with bars represent real‐time quantitative polymerase chain reaction (RT‐qPCR) analysis of transcript levels of *R. irregularis* marker gene, *ELONGATION FACTOR 1α* (*RiEF1α*) and *M. paleacea* marker genes associated with AMF colonisation: *STUNTED ARBUSCULE* (*MpaSTR*), *AMMONIUM TRANSPORTER 2* (*MpaAMT2*) and *SCARECROW (MpaSCR*), in *M. paleacea* thalli 8 wk after inoculation with *R. irregularis*. Each point represents a biological replicate with cDNA from at least two thalli. Transcript level was normalised to the geometric mean of the *M. paleacea* housekeeping genes *ADENINE PHOSPHORIBOSYL TRANSFERASE* (*MpaAPT*) and *MpaEF1α*. Bars and error bars indicate the mean and the SD, respectively. *n* ≥ 4. Kruskal–Wallis test with multiple comparisons against *WT::Cas9*. ***, *P* < 0.001; **, *P* < 0.01; *, *P* < 0.05. (c) Scatter dot plot showing the percentage of AMF thallus length colonisation in the indicated genotype: *n* = 10 (WT::*Cas9*), *n* = 10 (*Mpacngc1/3 #2*), *n* = 6 (*Mpacngc3/4 #7*), *n* = 10 (*Mpacngc1 #3*) and *n* = 10 (*Mpacngc3 #6*) from data presented in (a). Bars and error bars indicate the mean and the SD, respectively. Statistical analysis was performed using one‐way ANOVA followed by Tukey's multiple comparison test. Different letters indicate significant differences between groups.

In *M. truncatula*, DMI1‐dependent Ca^2+^ oscillations during AM symbiosis require three paralogous CNGC15s (Charpentier *et al*., 2016). In *M. paleacea*, our results demonstrate that *MpaCNGC1*, *MpaCNGC3* and *MpaCNGC4* are ubiquitously expressed throughout the thallus; however, loss of *MpaCNGC3* and *MpaCNGC4* alone is not sufficient to abolish AM fungal colonisation, which occurs in absence of rhizoids growth. These observations suggest that, if MpaCNGCs are required for AM symbiosis, this function may depend on the combined activity of more than two CNGCs. To test whether MpaCNGCs mediate AM symbiosis, we assessed AM fungal colonisation in single and higher‐order *Mpacngc* mutants. After 8 wk of inoculation with *R. irregularis*, thalli of *M. paleacea* WT::*Cas9* and all single *Mpacngc* mutants were fully colonised (Fig. 4a). Transcript levels of *R. irregularis ELONGATION FACTOR 1α* (*RiEF1α*; Gomez‐Gallego *et al*., 2019), together with the *M*. *paleacea* genes *STUNTED ARBUSCULE* (*MpaSTR*), *AMMONIUM TRANSPORTER 2* (*MpaAMT2*), and *SCARECROW* (*MpaSCR*), which are upregulated in arbuscule‐containing cells (Rich *et al*., 2021), were similar between individual *Mpacngc* mutants and WT::*Cas9* thalli after 8 wk of *R. irregularis* inoculation (Fig. 4a,b). Likewise, AM fungal colonisation was observed in the double mutants *Mpacngc1/3*, *Mpacngc1/4* and *Mpacngc3/4* (Fig. 4a). However, whereas 100% of *Mpacngc1/4* thalli were colonised, knockout of *MpaCNGC3* in combination with either *MpaCNGC1* or *MpaCNGC4* resulted in a reduction in both the proportion of colonised thalli and the extent of AM fungal colonisation within the midrib (Fig. 4a,c). This reduction was accompanied by a trend towards lower expression of *RiEF1α*, *MpaSCR* and *MpaAMT2*, although these differences were not statistically significant, with the exception of *MpaAMT2* expression in *Mpacngc3/4* (Fig. 4b). By contrast, no AM fungal colonisation was detected in the triple mutant *Mpacngc1/3/4 #2*, which was further confirmed by the absence of *RiEF1α*, *MpaSTR*, *MpaAMT2* and *MpaSCR* expression in *Mpacngc1/3/4 #30* (Fig. 4a,b).

**Fig. 5:**
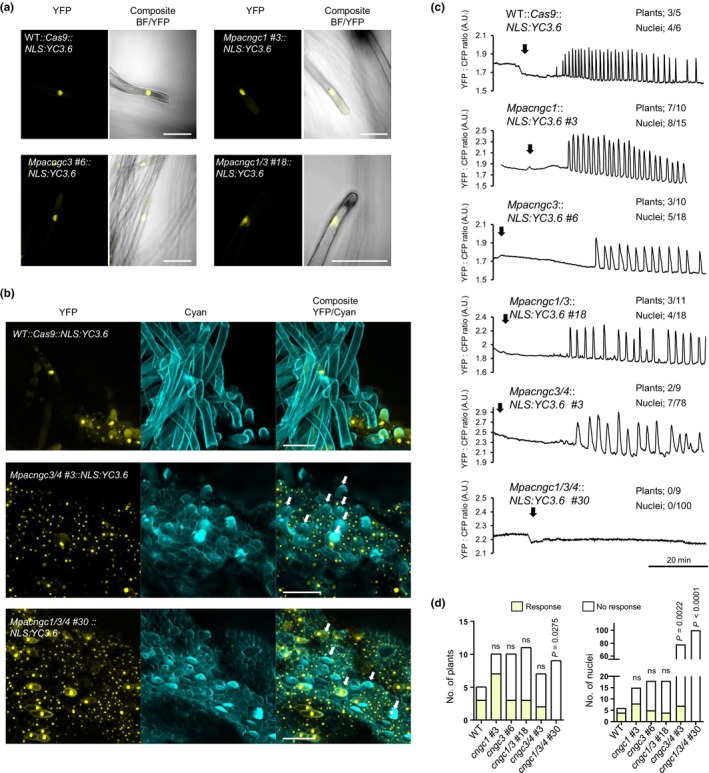
Germinated spore exudate‐induced nuclear calcium oscillation is abolished in *Mpacngc1/3/4* triple mutant. (a, b) Representative confocal laser scanning microscopy images of *Marchantia paleacea* rhizoids expressing the nuclear‐localised Ca^2+^ reporter *Yellow Cameleon v.3.6* (*NLS:YC3.6*), visualised in the yellow fluorescent protein (YFP) channel. Bars, 100 μm. (a) Rhizoids of wild‐type expressing *Cas9* (WT::*Cas9*::*NLS:YC3.6*), *Mpacngc1::NLS:YC3.6 #3*, *Mpacngc3::NLS:YC3.6 #6* and *Mpacngc1/3::NLS:YC3.6 #18*. Images are single confocal optical sections. Composite images show brightfield (BF) merged with the YFP channel. (b) Rhizoids of WT::*Cas9*::*NLS:YC3.6*, and bulged and nonbulged cells of *Mpacngc3/4::NLS:YC3.6 #3* and *Mpacngc1/3/4::NLS:YC3.6 #30*. The cyan channel shows cell walls stained with SR2200. White arrows indicate bulged cells. Images are maximum‐intensity projections of Z‐stacks. (c) Representative Ca^2+^ oscillation traces induced by *R. irregularis* germinated spore exudate (GSE) in *M. paleacea* WT::*Cas9*::*NLS:YC3.6* and *Mpacngc* mutants expressing *NLS:YC3.6*. All lines expressed *NLS:YC3.6* under the control of the *M. polymorpha EF1α* promoter. Traces show the ratio of YFP to cyan fluorescent protein (CFP) fluorescence in arbitrary units (A.U.). Arrowheads indicate the timing of GSE application. Numbers indicate the number of responding plants or nuclei relative to the total number analysed. Bar indicates 20 min. (d) Contingency bar chart showing the number of plants or nuclei classified as responding (yellow bars) and nonresponding (white bars) to GSE, based on nuclear Ca^2+^ oscillation data presented in (c). Statistical significance was assessed using Fisher's exact test. ns, not significant; *P‐*values are indicated.

To test whether *Mpacngc1/3/4* mutant is impaired in AM fungal germinated spore exudate (GSE)‐induced nuclear Ca^2+^ oscillations, knockout *Mpacngc* mutants expressing the nuclear localised Ca^2+^ reporter, *Yellow Cameleon 3.6* fused to the nuclear localisation signal (*NLS:YC3.6*) were tested for symbiosis‐mediated nuclear Ca^2+^ oscillations (Fig. 5a,b). Application of GSE induced nuclear Ca^2+^ oscillation in nuclei of rhizoids of the WT, *Mpacngc1*, *Mpacngc3* and *Mpacngc1/3* mutants, as well as in both bulged and nonbulged cells of the *Mpacngc3/4* mutants, with 28% of nonbulged cells responding compared with only 9% of bulged cells (Figs 5c,d, S6). Consistent with the absence of AM fungal colonisation, nuclear calcium oscillations were not detected in either bulged or nonbulged cells of the *Mpacngc1/3/4* mutant following GSE application (Figs 5c,d, S6). Together, these results demonstrate that MpaCNGC1, MpCNGC3 and MpaCNGC4 are collectively required for the generation of endosymbiosis‐associated nuclear Ca^2+^ oscillations and for successful AM fungal colonisation. Moreover, the presence of GSE‐induced nuclear Ca^2+^ oscillations and AM fungal colonisation in both bulged and nonbulged cells of *Mpacngc3/4* mutants reveals that rhizoid growth is uncoupled from AM symbiosis signalling and fungal colonisation in *M. paleacea*.

## Discussion

By integrating phylogenetic analysis with higher‐order mutant analysis and nuclear Ca^2+^ imaging, we show that CNGCs are essential for endosymbiosis‐associated nuclear Ca^2+^ oscillations and AM fungal colonisation, while rhizoid outgrowth itself is dispensable for fungal entry. These findings deepen our understanding of the evolutionary origin of symbiotic Ca^2+^ signalling and demonstrate that rhizoid infection can be uncoupled from symbiotic competence in *M. paleacea*.

Phylogenetic analysis across streptophytes resolves land plant CNGCs into three ancient superclades (A–C), indicating that the major diversification of this family predates both land plant evolution and AM symbiosis. Within this framework, MpaCNGC1 and MpaCNGC3 are sister to the angiosperm lineage containing MtCNGC15a/b/c, which are required for symbiosis‐associated Ca^2+^ oscillations in *M. truncatula*. In *M. paleacea*, however, simultaneous loss of *MpaCNGC1*, *MpaCNGC3* and *MpaCNGC4* is required to completely abolish symbiosis‐induced nuclear Ca^2+^ oscillations and blocks AM fungal colonisation. Notably, these three CNGCs belong to two distinct superclades, indicating that in bryophytes the symbiotic function of CNGCs is not constrained by phylogenetic grouping. These results further demonstrate that endosymbiosis signalling represents a conserved function of CNGCs in land plants.

In addition to their role in endosymbiosis, both MpaCNGC3 (superclade C) and MpaCNGC4 (superclade A) promote rhizoid elongation, revealing a conserved requirement for CNGCs in anisotropic cell expansion. This parallels the involvement of CNGCs in pollen tube and root hair growth in angiosperms and is consistent with the reliance of tip‐growing cells on oscillatory Ca^2+^ dynamics (Bibikova *et al*., 1997; Damineli *et al*., 2017; Bascom Jr. *et al*., 2018). Among angiosperm CNGCs, AtCNGC7, AtCNGC8 and AtCNGC18 are required for pollen tube development (Frietsch *et al*., 2007; Tunc‐Ozdemir *et al*., 2013; Gao *et al*., 2016), whereas AtCNGC5, AtCNGC6, AtCNGC9 and AtCNGC14 contribute to root hair growth (Brost *et al*., 2019; Tan *et al*., 2020). The *Atcngc5/6/9* triple and quadruple mutant *Atcngc5/6/9/14* exhibits reduced root hair length and increased branching when grown on the surface of vertical media or in media. However, when grown in media, increased root hair bursting is observed only in the *Atcngc5/6/9/14* quadruple mutant and *Atcngc14* single mutant, indicating that AtCNGC14 specifically contributes to root hair integrity in response to mechanical or physical constraints. By contrast, AtCNGC5, AtCNGC6 and AtCNGC9 primarily promote root hair elongation (Brost *et al*., 2019; Tan *et al*., 2020). AtCNGC5/6/7/8/9/14/18 and MpaCNGC3 belong to superclade C, while MpaCNGC4 belongs to superclade A, indicating that the anisotropic growth function of CNGCs is not constrained by phylogenetic grouping in *Marchantia*. In this lineage, limited gene expansion may have restricted subfunctionalisation, resulting in the retention of overlapping functions among CNGCs from different clades. By contrast, expansion of this gene family in flowering plants has likely promoted functional partitioning, restricting this role to a single clade.

The conserved role of CNGC in anisotropic cell expansion of both rhizoid and root hair cells aligns with extensive conservation between rhizoid and root hair developmental programmes (Honkanen *et al*., 2016). In *M. polymorpha*, the NADPH oxidase MpRBOH1 is essential for rhizoid formation (Chu *et al*., 2023), which is reminiscent of the role of AtRBOHC/RHD2 in root hair growth (Foreman *et al*., 2003). MpRBOH1 contains two EF‐hand motifs in its cytosolic N‐terminus, and Ca^2+^ binding to these EF‐hands is required for reactive oxygen species (ROS) production *in planta* (Hashimoto *et al*., 2023). In this context, Ca^2+^ signals mediated by MpCNGC3/4 may promote rhizoid growth by activating RBOH‐dependent ROS production. Beyond regulation of ROS production, Ca^2+^ signalling may also influence membrane trafficking (Himschoot *et al*., 2017), cytoskeleton dynamics (Himschoot *et al*., 2015) and ROP‐based polarity establishment (Mulvey & Dolan, 2023), offering multiple nonmutually exclusive routes through which CNGCs could support rhizoid growth.

Strikingly, despite the severe rhizoid defects of the *Mpacngc3/4* mutant, AMF still penetrate lower epidermal cells (both bulged and nonbulged), colonise the thallus midrib and form arbuscules. Consistently, both bulged and nonbulged cells generate nuclear Ca^2+^ oscillations in response to GSE. This demonstrates that rhizoids, while representing a major entry route, are not strictly required for AM symbiosis in *M. paleacea*. This flexibility in infection strategy is further supported by *in vitro* studies in the hornwort *Anthoceros punctatus* and liverwort *Plagiochasma rupestre*, where colonisation is observed through nonrhizoid epidermal cells (Silvani *et al*., 2012). The dispensability of tip‐growing cells for fungal entry closely parallels observations in flowering plants, where AMF infect both trichoblast and atrichoblast epidermal cells. Together, these findings indicate that flexible epidermal entry is an evolutionarily conserved feature of AM symbiosis that predates the origin of true roots. These results further reveal the conserved involvement of CNGCs in facilitating nutrient acquisition by coordinated rhizoid development and AM symbiosis during terrestrialisation.

Together, our findings show that a reduced CNGC repertoire in bryophyte underpins multiple biological processes through combinatorial channel activity. This functional versatility underscores the central role of Ca^2+^ signalling as an integrative regulatory mechanism that supported developmental innovation and symbiotic competence during land plant evolution.

## Competing interests

None declared.

## Author contributions

MC conceived and supervised the project. AHCL performed the majority of the work. AC contributed to the molecular cloning and *M. paleacea CNGC* promoter analysis. JR performed Cryo‐scanning electron microscopy. AHCL and MC analysed data. MC wrote the manuscript with AHCL input. All authors agreed on the manuscript before submission.

## Disclaimer

The New Phytologist Foundation remains neutral with regard to jurisdictional claims in maps and in any institutional affiliations.

## Supporting information


**Fig. S1** *Marchantia paleacea* contains two co‐orthologues to the angiosperm Group I, II and III CNGCs.
**Fig. S2** Expression analysis of *MpaCNGC* promoters.
**Fig. S3** Generation of *Mpacngc* CRISPR/Cas9 knockout mutants.
**Fig. S4** Truncation of MpaCNGC proteins in CRISPR/Cas9 knockout mutants.
**Fig. S5** *MpaCNGC3* and *MpaCNGC4* are required for thallus development.
**Fig. S6** Nonbulged cells in the *Mpacngc1/3/4* mutant are impaired in germinated spore exudate‐induced nuclear Ca^2+^ oscillations.

**Movie S1** 3D reconstruction of a *Mpacngc3/4 #3* plant colonised by *Rhizophagus irregularis* through a bulged cell.

**Movie S2** 3D reconstruction of a *Mpacngc3/4 #3* plant colonised by *Rhizophagus irregularis* through a nonbulged cell.
**Table S1** List of sequence accessions used for phylogenetic analysis.
**Table S2** List of primers used in this study.
**Table S3** List of Level 1 and Level 2 Golden Gate vectors generated in this study.
**Table S4** List of accession numbers for *Marchantia* genes used in this study.
Please note: Wiley is not responsible for the content or functionality of any Supporting Information supplied by the authors. Any queries (other than missing material) should be directed to the *New Phytologist* Central Office.

## Data Availability

All data are available in the public repositories listed in the Materials and Methods section. All accession numbers are provided in Tables S1 and S4.

## References

[nph71296-bib-0001] Ane JM , Kiss GB , Riely BK , Penmetsa RV , Oldroyd GE , Ayax C , Levy J , Debelle F , Baek JM , Kalo P *et al*. 2004. *Medicago truncatula* DMI1 required for bacterial and fungal symbioses in legumes. Science 303: 1364–1367. 14963334 10.1126/science.1092986

[nph71296-bib-0002] Bascom CS Jr , Hepler PK , Bezanilla M . 2018. Interplay between ions, the cytoskeleton, and cell wall properties during tip growth. Plant Physiology 176: 28–40. 29138353 10.1104/pp.17.01466 PMC5761822

[nph71296-bib-0003] Bibikova TN , Zhigilei A , Gilroy S . 1997. Root hair growth in *Arabidopsis thaliana* is directed by calcium and an endogenous polarity. Planta 203: 495–505. 9421933 10.1007/s004250050219

[nph71296-bib-0004] Bonfante P , Genre A . 2010. Mechanisms underlying beneficial plant‐fungus interactions in mycorrhizal symbiosis. Nature Communications 1: 48. 10.1038/ncomms1046 20975705

[nph71296-bib-0005] Bowman JL , Arteaga‐Vazquez M , Berger F , Briginshaw LN , Carella P , Aguilar‐Cruz A , Davies KM , Dierschke T , Dolan L , Dorantes‐Acosta AE *et al*. 2022. The renaissance and enlightenment of *Marchantia* as a model system. Plant Cell 34: 3512–3542. 35976122 10.1093/plcell/koac219 PMC9516144

[nph71296-bib-0006] Brost C , Studtrucker T , Reimann R , Denninger P , Czekalla J , Krebs M , Fabry B , Schumacher K , Grossmann G , Dietrich P . 2019. Multiple cyclic nucleotide‐gated channels coordinate calcium oscillations and polar growth of root hairs. The Plant Journal 99: 910–923. 31033043 10.1111/tpj.14371

[nph71296-bib-0007] Brundrett MC , Tedersoo L . 2018. Evolutionary history of mycorrhizal symbioses and global host plant diversity. New Phytologist 220: 1108–1115. 29355963 10.1111/nph.14976

[nph71296-bib-0008] Camacho C , Coulouris G , Avagyan V , Ma N , Papadopoulos J , Bealer K , Madden TL . 2009. Blast+: architecture and applications. BMC Bioinformatics 10: 421. 20003500 10.1186/1471-2105-10-421 PMC2803857

[nph71296-bib-0009] Carpenter EJ , Matasci N , Ayyampalayam S , Wu S , Sun J , Yu J , Jimenez Vieira FR , Bowler C , Dorrell RG , Gitzendanner MA *et al*. 2019. Access to RNA‐sequencing data from 1,173 plant species: the 1000 plant transcriptomes initiative (1KP). GigaScience 8: giz126. 31644802 10.1093/gigascience/giz126 PMC6808545

[nph71296-bib-0010] Chari R , Yeo NC , Chavez A , Church GM . 2017. sgRNA scorer 2.0: a species‐independent model to predict CRISPR/Cas9 activity. ACS Synthetic Biology 6: 902–904. 28146356 10.1021/acssynbio.6b00343 PMC5793212

[nph71296-bib-0011] Charpentier M , Sun J , Vaz Martins T , Radhakrishnan GV , Findlay K , Soumpourou E , Thouin J , Very AA , Sanders D , Morris RJ *et al*. 2016. Nuclear‐localized cyclic nucleotide‐gated channels mediate symbiotic calcium oscillations. Science 352: 1102–1105. 27230377 10.1126/science.aae0109

[nph71296-bib-0012] Chu J , Monte I , DeFalco TA , Koster P , Derbyshire P , Menke FLH , Zipfel C . 2023. Conservation of the PBL‐RBOH immune module in land plants. Current Biology 33: 1130–1137. 36796360 10.1016/j.cub.2023.01.050

[nph71296-bib-0013] Cook NM , Gobbato G , Jacott CN , Marchal C , Hsieh CY , Lam AHC , Simmonds J , Del Cerro P , Gomez PN , Rodney C *et al*. 2025. Autoactive CNGC15 enhances root endosymbiosis in legume and wheat. Nature 638: 752–759. 39814887 10.1038/s41586-024-08424-7 PMC11839481

[nph71296-bib-0014] Damineli DSC , Portes MT , Feijo JA . 2017. Oscillatory signatures underlie growth regimes in Arabidopsis pollen tubes: computational methods to estimate tip location, periodicity, and synchronization in growing cells. Journal of Experimental Botany 68: 3267–3281. 28369603 10.1093/jxb/erx032 PMC5853864

[nph71296-bib-0015] DeFalco TA , Moeder W , Yoshioka K . 2016. Opening the gates: insights into cyclic nucleotide‐gated channel‐mediated signaling. Trends in Plant Science 21: 903–906. 27623305 10.1016/j.tplants.2016.08.011

[nph71296-bib-0016] Del Cerro P , Cook NM , Huisman R , Dangeville P , Grubb LE , Marchal C , Ho Ching Lam A , Charpentier M . 2022. Engineered CaM2 modulates nuclear calcium oscillation and enhances legume root nodule symbiosis. Proceedings of the National Academy of Sciences, USA 119: e2200099119. 10.1073/pnas.2200099119 PMC9060481 35324326

[nph71296-bib-0017] Desiro A , Duckett JG , Pressel S , Villarreal JC , Bidartondo MI . 2013. Fungal symbioses in hornworts: a chequered history. Proceedings of the Biological Sciences 280: 20130207. 23536598 10.1098/rspb.2013.0207 PMC3619511

[nph71296-bib-0018] Engler C , Gruetzner R , Kandzia R , Marillonnet S . 2009. Golden gate shuffling: a one‐pot DNA shuffling method based on type IIs restriction enzymes. PLoS ONE 4: e5553. 19436741 10.1371/journal.pone.0005553 PMC2677662

[nph71296-bib-0019] Feng F , Sun J , Radhakrishnan GV , Lee T , Bozsoki Z , Fort S , Gavrin A , Gysel K , Thygesen MB , Andersen KR *et al*. 2019. A combination of chitooligosaccharide and lipochitooligosaccharide recognition promotes arbuscular mycorrhizal associations in *Medicago truncatula* . Nature Communications 10: 5047. 10.1038/s41467-019-12999-5 PMC6834629 31695035

[nph71296-bib-0020] Fonseca H , Berbara RLL , Pereira ML . 2006. *Lunularia cruciata*, a potential *in vitro* host for *Glomus proliferum* and *G. intraradices* . Mycorrhiza 16: 503–508. 16896799 10.1007/s00572-006-0061-x

[nph71296-bib-0021] Foreman J , Demidchik V , Bothwell JH , Mylona P , Miedema H , Torres MA , Linstead P , Costa S , Brownlee C , Jones JD *et al*. 2003. Reactive oxygen species produced by NADPH oxidase regulate plant cell growth. Nature 422: 442–446. 12660786 10.1038/nature01485

[nph71296-bib-0022] Frietsch S , Wang YF , Sladek C , Poulsen LR , Romanowsky SM , Schroeder JI , Harper JF . 2007. A cyclic nucleotide‐gated channel is essential for polarized tip growth of pollen. Proceedings of the National Academy of Sciences, USA 104: 14531–14536. 10.1073/pnas.0701781104 PMC1964830 17726111

[nph71296-bib-0023] Gao QF , Gu LL , Wang HQ , Fei CF , Fang X , Hussain J , Sun SJ , Dong JY , Liu H , Wang YF . 2016. Cyclic nucleotide‐gated channel 18 is an essential Ca^2+^ channel in pollen tube tips for pollen tube guidance to ovules in Arabidopsis. Proceedings of the National Academy of Sciences, USA 113: 3096–3101. 10.1073/pnas.1524629113 PMC4801260 26929345

[nph71296-bib-0024] Genre A , Chabaud M , Balzergue C , Puech‐Pages V , Novero M , Rey T , Fournier J , Rochange S , Becard G , Bonfante P *et al*. 2013. Short‐chain chitin oligomers from arbuscular mycorrhizal fungi trigger nuclear Ca^2+^ spiking in *Medicago truncatula* roots and their production is enhanced by strigolactone. New Phytologist 198: 190–202. 23384011 10.1111/nph.12146

[nph71296-bib-0025] Gomez‐Gallego T , Benabdellah K , Merlos MA , Jimenez‐Jimenez AM , Alcon C , Berthomieu P , Ferrol N . 2019. The *Rhizophagus irregularis* genome encodes two CTR copper transporters that mediate Cu import into the cytosol and a CTR‐like protein likely involved in copper tolerance. Frontiers in Plant Science 10: 604. 31156674 10.3389/fpls.2019.00604 PMC6531763

[nph71296-bib-0026] Goodstein DM , Shu S , Howson R , Neupane R , Hayes RD , Fazo J , Mitros T , Dirks W , Hellsten U , Putnam N *et al*. 2012. Phytozome: a comparative platform for green plant genomics. Nucleic Acids Research 40: D1178–D1186. 22110026 10.1093/nar/gkr944 PMC3245001

[nph71296-bib-0027] Govindarajulu M , Pfeffer PE , Jin H , Abubaker J , Douds DD , Allen JW , Bucking H , Lammers PJ , Shachar‐Hill Y . 2005. Nitrogen transfer in the arbuscular mycorrhizal symbiosis. Nature 435: 819–823. 15944705 10.1038/nature03610

[nph71296-bib-0028] Guinel FC , Hirsch AM . 2000. The involvement of root hairs in mycorrhizal associations. Tokyo, Japan: Springer.

[nph71296-bib-0029] Hashimoto T , Hashimoto K , Shindo H , Tsuboyama S , Miyakawa T , Tanokura M , Kuchitsu K . 2023. Enhanced Ca(2+) binding to EF‐hands through phosphorylation of conserved serine residues activates MpRBOHB and chitin‐triggered ROS production. Physiologia Plantarum 175: e14101. 38148249 10.1111/ppl.14101

[nph71296-bib-0030] He J , Zhang C , Dai H , Liu H , Zhang X , Yang J , Chen X , Zhu Y , Wang D , Qi X *et al*. 2019. A LysM receptor heteromer mediates perception of arbuscular mycorrhizal symbiotic signal in rice. Molecular Plant 12: 1561–1576. 31706032 10.1016/j.molp.2019.10.015

[nph71296-bib-0031] Himschoot E , Beeckman T , Friml J , Vanneste S . 2015. Calcium is an organizer of cell polarity in plants. Biochimica et Biophysica Acta 1853: 2168–2172. 25725133 10.1016/j.bbamcr.2015.02.017

[nph71296-bib-0032] Himschoot E , Pleskot R , Van Damme D , Vanneste S . 2017. The ins and outs of Ca(2+) in plant endomembrane trafficking. Current Opinion in Plant Biology 40: 131–137. 28965016 10.1016/j.pbi.2017.09.003

[nph71296-bib-0033] Hoang DT , Chernomor O , von Haeseler A , Minh BQ , Vinh LS . 2018. UFBoot2: improving the ultrafast bootstrap approximation. Molecular Biology and Evolution 35: 518–522. 29077904 10.1093/molbev/msx281 PMC5850222

[nph71296-bib-0034] Honkanen S , Jones VAS , Morieri G , Champion C , Hetherington AJ , Kelly S , Proust H , Saint‐Marcoux D , Prescott H , Dolan L . 2016. The mechanism forming the cell surface of tip‐growing rooting cells is conserved among land plants. Current Biology 26: 3238–3244. 27866889 10.1016/j.cub.2016.09.062 PMC5154754

[nph71296-bib-0035] Hu R , Li X , Hu Y , Zhang R , Lv Q , Zhang M , Sheng X , Zhao F , Chen Z , Ding Y *et al*. 2023. Adaptive evolution of the enigmatic Takakia now facing climate change in Tibet. Cell 186: 3558–3576. 37562403 10.1016/j.cell.2023.07.003

[nph71296-bib-0036] Humphreys CP , Franks PJ , Rees M , Bidartondo MI , Leake JR , Beerling DJ . 2010. Mutualistic mycorrhiza‐like symbiosis in the most ancient group of land plants. Nature Communications 1: 103. 10.1038/ncomms1105 21045821

[nph71296-bib-0037] Jones VA , Dolan L . 2012. The evolution of root hairs and rhizoids. Annals of Botany 110: 205–212. 22730024 10.1093/aob/mcs136 PMC3394659

[nph71296-bib-0038] Kalyaanamoorthy S , Minh BQ , Wong TKF , von Haeseler A , Jermiin LS . 2017. ModelFinder: fast model selection for accurate phylogenetic estimates. Nature Methods 14: 587–589. 28481363 10.1038/nmeth.4285 PMC5453245

[nph71296-bib-0039] Kanno S , Fukumura H , Sato S , Moriya KC , Sakai Y , Ishizaki K . 2026. Rhizoid‐mediated phosphate uptake and internal transport in the non‐vascular plant *Marchantia polymorpha* . New Phytologist 250: 708–716. 41740965 10.1111/nph.70980

[nph71296-bib-0040] Katoh K , Standley DM . 2013. Mafft multiple sequence alignment software version 7: improvements in performance and usability. Molecular Biology and Evolution 30: 772–780. 23329690 10.1093/molbev/mst010 PMC3603318

[nph71296-bib-0041] Kawamura S , Romani F , Yagura M , Mochizuki T , Sakamoto M , Yamaoka S , Nishihama R , Nakamura Y , Yamato KT , Bowman JL *et al*. 2022. MarpolBase expression: a web‐based, comprehensive platform for visualization and analysis of transcriptomes in the liverwort *Marchantia polymorpha* . Plant & Cell Physiology 63: 1745–1755. 36083565 10.1093/pcp/pcac129 PMC9680858

[nph71296-bib-0042] Keymer A , Pimprikar P , Wewer V , Huber C , Brands M , Bucerius SL , Delaux PM , Klingl V , Ropenack‐Lahaye EV , Wang TL *et al*. 2017. Lipid transfer from plants to arbuscular mycorrhiza fungi. eLife 6: e29107. 28726631 10.7554/eLife.29107 PMC5559270

[nph71296-bib-0043] Kim D , Paggi JM , Park C , Bennett C , Salzberg SL . 2019. Graph‐based genome alignment and genotyping with HISAT2 and HISAT‐genotype. Nature Biotechnology 37: 907–915. 10.1038/s41587-019-0201-4 PMC7605509 31375807

[nph71296-bib-0044] Kobae Y , Ohtomo R , Morimoto S , Sato D , Nakagawa T , Oka N , Sato S . 2019. Isolation of native arbuscular mycorrhizal fungi within young thalli of the liverwort *Marchantia paleacea* . Plants (Basel) 8: e29107. 10.3390/plants8060142 PMC6631804 31151150

[nph71296-bib-0045] Krawczyk K , Szablinska‐Piernik J , Paukszto L , Mazdziarz M , Sulima P , Przyborowski JA , Szczecinska M , Sawicki J . 2025. Chromosome‐scale telomere to telomere genome assembly of common crystalwort (*Riccia sorocarpa* Bisch.). Sci Data 12: 77. 39814758 10.1038/s41597-025-04373-6 PMC11735767

[nph71296-bib-0046] Lam AHC , Cooke A , Wright H , Lawson DM , Charpentier M . 2024. Evolution of endosymbiosis‐mediated nuclear calcium signaling in land plants. Current Biology 34: 2212–2220.e7. 38642549 10.1016/j.cub.2024.03.063

[nph71296-bib-0047] Leebens‐Mack James H , Barker Michael S , Carpenter Eric J , Deyholos Michael K , Gitzendanner Matthew A , Graham Sean W , Ivo G , Zheng L , Michael M , Siavash M *et al*. 2019. One thousand plant transcriptomes and the phylogenomics of green plants. Nature 574: 679–685. 31645766 10.1038/s41586-019-1693-2 PMC6872490

[nph71296-bib-0048] Letunic I , Bork P . 2024. Interactive Tree of Life (iTOL) v.6: recent updates to the phylogenetic tree display and annotation tool. Nucleic Acids Research 52: W78–W82. 38613393 10.1093/nar/gkae268 PMC11223838

[nph71296-bib-0049] Li H , Handsaker B , Wysoker A , Fennell T , Ruan J , Homer N , Marth G , Abecasis G , Durbin R , Genome Project Data Processing S . 2009. The sequence alignment/map format and SAMtools . Bioinformatics 25: 2078–2079. 19505943 10.1093/bioinformatics/btp352 PMC2723002

[nph71296-bib-0050] Ligrone R , Carafa A , Lumini E , Bianciotto V , Bonfante P , Duckett JG . 2007. Glomeromycotean associations in liverworts: a molecular, cellular, and taxonomic analysis. American Journal of Botany 94: 1756–1777. 21636371 10.3732/ajb.94.11.1756

[nph71296-bib-0051] Linde AM , Singh S , Bowman JL , Eklund M , Cronberg N , Lagercrantz U . 2023. Genome evolution in plants: complex thalloid liverworts (Marchantiopsida). Genome Biology and Evolution 15: evad014. 36726237 10.1093/gbe/evad014 PMC9985172

[nph71296-bib-0052] Love MI , Huber W , Anders S . 2014. Moderated estimation of fold change and dispersion for RNA‐seq data with DESeq2. Genome Biology 15: 550. 25516281 10.1186/s13059-014-0550-8 PMC4302049

[nph71296-bib-0053] Luginbuehl LH , Menard GN , Kurup S , Van Erp H , Radhakrishnan GV , Breakspear A , Oldroyd GED , Eastmond PJ . 2017. Fatty acids in arbuscular mycorrhizal fungi are synthesized by the host plant. Science 356: 1175–1178. 28596311 10.1126/science.aan0081

[nph71296-bib-0086] Marchetti F , Cainzos M , Cascallares M , Distéfano AM , Setzes N , López GA , Zabaleta E , Pagnussat GC . 2021. Heat stress in Marchantia polymorpha: sensing and mechanisms underlying a dynamic response. Plant, Cell & Environment 44: 2134–2149. 10.1111/pce.13914 33058168

[nph71296-bib-0054] Maser P , Thomine S , Schroeder JI , Ward JM , Hirschi K , Sze H , Talke IN , Amtmann A , Maathuis FJ , Sanders D *et al*. 2001. Phylogenetic relationships within cation transporter families of Arabidopsis. Plant Physiology 126: 1646–1667. 11500563 10.1104/pp.126.4.1646 PMC117164

[nph71296-bib-0055] Meijering E , Jacob M , Sarria JC , Steiner P , Hirling H , Unser M . 2004. Design and validation of a tool for neurite tracing and analysis in fluorescence microscopy images. Cytometry. Part A 58: 167–176. 10.1002/cyto.a.20022 15057970

[nph71296-bib-0056] Menand B , Yi K , Jouannic S , Hoffmann L , Ryan E , Linstead P , Schaefer DG , Dolan L . 2007. An ancient mechanism controls the development of cells with a rooting function in land plants. Science 316: 1477–1480. 17556585 10.1126/science.1142618

[nph71296-bib-0057] Ming Y , Peng Y , Liu Q , Fu D , Lin Q , You P , Wang X , Zhang X , Wang Y , Gong Z *et al*. 2025. Coordinated control of calcium signaling by CPK3 and CaM2 via CNGCs in response to cold stress in Arabidopsis. Developmental Cell 60: 3222–3235. 40633536 10.1016/j.devcel.2025.06.020

[nph71296-bib-0058] Montgomery SA , Tanizawa Y , Galik B , Wang N , Ito T , Mochizuki T , Akimcheva S , Bowman JL , Cognat V , Marechal‐Drouard L *et al*. 2020. Chromatin organization in early land plants reveals an ancestral association between H3K27me3, transposons, and constitutive heterochromatin. Current Biology 30: 573–588. 32004456 10.1016/j.cub.2019.12.015 PMC7209395

[nph71296-bib-0059] Mulvey H , Dolan L . 2023. RHO GTPase of plants regulates polarized cell growth and cell division orientation during morphogenesis. Current Biology 33: 2897–2911. 37385256 10.1016/j.cub.2023.06.015

[nph71296-bib-0060] Nguyen LT , Schmidt HA , von Haeseler A , Minh BQ . 2015. IQ‐Tree: a fast and effective stochastic algorithm for estimating maximum‐likelihood phylogenies. Molecular Biology and Evolution 32: 268–274. 25371430 10.1093/molbev/msu300 PMC4271533

[nph71296-bib-0061] Novero M , A , Genre KS , Bonfante P . 2008. Root hair colonization by mycorrhizal fungi. Berlin, Germany: Springer.

[nph71296-bib-0062] Pertea M , Pertea GM , Antonescu CM , Chang TC , Mendell JT , Salzberg SL . 2015. StringTie enables improved reconstruction of a transcriptome from RNA‐seq reads. Nature Biotechnology 33: 290–295. 10.1038/nbt.3122 PMC4643835 25690850

[nph71296-bib-0063] Proust H , Honkanen S , Jones VA , Morieri G , Prescott H , Kelly S , Ishizaki K , Kohchi T , Dolan L . 2016. RSL class I genes controlled the development of epidermal structures in the common ancestor of land plants. Current Biology 26: 93–99. 26725198 10.1016/j.cub.2015.11.042 PMC4712171

[nph71296-bib-0064] Remy W , Taylor TN , Hass H , Kerp H . 1994. Four hundred‐million‐year‐old vesicular arbuscular mycorrhizae. Proceedings of the National Academy of Sciences, USA 91: 11841–11843. 10.1073/pnas.91.25.11841 PMC45331 11607500

[nph71296-bib-0065] Rich MK , Vigneron N , Libourel C , Keller J , Xue L , Hajheidari M , Radhakrishnan GV , Le Ru A , Diop SI , Potente G *et al*. 2021. Lipid exchanges drove the evolution of mutualism during plant terrestrialization. Science 372: 864–868. 34016782 10.1126/science.abg0929

[nph71296-bib-0066] Rimington WR , Pressel S , Duckett JG , Field KJ , Read DJ , Bidartondo MI . 2018. Ancient plants with ancient fungi: liverworts associate with early‐diverging arbuscular mycorrhizal fungi. Proceedings of the Biological Sciences 285: 20181600. 30305437 10.1098/rspb.2018.1600 PMC6191707

[nph71296-bib-0067] Schafran P , Hauser DA , Nelson JM , Xu X , Mueller LA , Kulshrestha S , Smalley I , de Vries S , Irisarri I , de Vries J *et al*. 2025. Pan‐phylum genomes of hornworts reveal conserved autosomes but dynamic accessory and sex chromosomes. Nature Plants 11: 49–62. 39753957 10.1038/s41477-024-01883-w

[nph71296-bib-0068] Schindelin J , Arganda‐Carreras I , Frise E , Kaynig V , Longair M , Pietzsch T , Preibisch S , Rueden C , Saalfeld S , Schmid B *et al*. 2012. Fiji: an open‐source platform for biological‐image analysis. Nature Methods 9: 676–682. 22743772 10.1038/nmeth.2019 PMC3855844

[nph71296-bib-0069] Schmittgen TD , Livak KJ . 2008. Analyzing real‐time PCR data by the comparative C(T) method. Nature Protocols 3: 1101–1108. 18546601 10.1038/nprot.2008.73

[nph71296-bib-0070] Silvani VA , Rothen CP , Rodriguez MA , Godeas A , Fracchia S . 2012. The thalloid liverwort *Plagiochasma rupestre* supports arbuscular mycorrhiza‐like symbiosis *in vitro* . World Journal of Microbiology and Biotechnology 28: 3393–3397. 22886707 10.1007/s11274-012-1146-7

[nph71296-bib-0071] Smith SE , Read DJ . 2008. Mycorrhizal symbiosis. New York, NY, USA: Academic Press.

[nph71296-bib-0072] Szablinska‐Piernik J , Sulima P , Sawicki J . 2026. Giant chromosomes of a tiny plant‐the complete telomere‐to‐telomere genome assembly of the simple thalloid liverwort *Apopellia endiviifolia* (Jungermanniopsida, Marchantiophyta). GigaScience 15: giaf145. 41316987 10.1093/gigascience/giaf145 PMC12885004

[nph71296-bib-0073] Tan QW , Lim PK , Chen Z , Pasha A , Provart N , Arend M , Nikoloski Z , Mutwil M . 2023. Cross‐stress gene expression atlas of *Marchantia polymorpha* reveals the hierarchy and regulatory principles of abiotic stress responses. Nature Communications 14: 986. 10.1038/s41467-023-36517-w PMC9946954 36813788

[nph71296-bib-0074] Tan X , Wang D , Zhang X , Zheng S , Jia X , Liu H , Liu Z , Yang H , Dai H , Chen X *et al*. 2025. A pair of LysM receptors mediates symbiosis and immunity discrimination in *Marchantia* . Cell 188: 1330–1348. 39855200 10.1016/j.cell.2024.12.024

[nph71296-bib-0075] Tan YQ , Yang Y , Zhang A , Fei CF , Gu LL , Sun SJ , Xu W , Wang L , Liu H , Wang YF . 2020. Three CNGC family members, CNGC5, CNGC6, and CNGC9, are required for constitutive growth of Arabidopsis root hairs as Ca(2+)‐permeable channels. Plant Communications 1: 100001. 33404548 10.1016/j.xplc.2019.100001 PMC7748020

[nph71296-bib-0076] Tan YY , Huang GW , Fan HY , Wu T , Guan ZL , Liu KD . 2024. CNGC20 plays dual roles in regulating plant growth and immunity in *Brassica napus* . Crop Journal 12: 1533–1546.

[nph71296-bib-0077] Teyssier E , Grat S , Landry D , Ouradou M , Rich MK , Fort S , Keller J , Lefebvre B , Delaux PM , Mbengue M . 2025. A plant lysin motif receptor‐like kinase plays an ancestral function in mycorrhiza. Proceedings of the National Academy of Sciences, USA 122: e2426063122. 10.1073/pnas.2426063122 PMC12184373 40498450

[nph71296-bib-0078] Tipper E , Leitao N , Dangeville P , Lawson DM , Charpentier M . 2023. A novel mutant allele of AtCNGC15 reveals a dual function of nuclear calcium release in the root meristem. Journal of Experimental Botany 74: 2572–2584. 36715622 10.1093/jxb/erad041 PMC10112680

[nph71296-bib-0079] Tunc‐Ozdemir M , Rato C , Brown E , Rogers S , Mooneyham A , Frietsch S , Myers CT , Poulsen LR , Malho R , Harper JF . 2013. Cyclic nucleotide gated channels 7 and 8 are essential for male reproductive fertility. PLoS ONE 8: e55277. 23424627 10.1371/journal.pone.0055277 PMC3570425

[nph71296-bib-0080] Wang W , Shi J , Xie Q , Jiang Y , Yu N , Wang E . 2017. Nutrient exchange and regulation in arbuscular mycorrhizal symbiosis. Molecular Plant 10: 1147–1158. 28782719 10.1016/j.molp.2017.07.012

[nph71296-bib-0081] Weber E , Engler C , Gruetzner R , Werner S , Marillonnet S . 2011. A modular cloning system for standardized assembly of multigene constructs. PLoS ONE 6: e16765. 21364738 10.1371/journal.pone.0016765 PMC3041749

[nph71296-bib-0082] Wheeler GL , Brownlee C . 2008. Ca^2+^ signalling in plants and green algae–changing channels. Trends in Plant Science 13: 506–514. 18703378 10.1016/j.tplants.2008.06.004

[nph71296-bib-0083] Yu J , Cai Y , Zhu Y , Zeng Y , Dong S , Zhang K , Wang S , Li L , Goffinet B , Liu H *et al*. 2022. Chromosome‐level genome assemblies of two Hypnales (mosses) reveal high intergeneric synteny. Genome Biology and Evolution 14: evac020. 35166770 10.1093/gbe/evac020 PMC8859630

[nph71296-bib-0084] Zhang J , Sun J , Chiu CH , Landry D , Li K , Wen J , Mysore KS , Fort S , Lefebvre B , Oldroyd GED *et al*. 2024. A receptor required for chitin perception facilitates arbuscular mycorrhizal associations and distinguishes root symbiosis from immunity. Current Biology 34: 1705–1717. 38574729 10.1016/j.cub.2024.03.015 PMC11037463

[nph71296-bib-0085] Zhou X , Peng T , Zeng Y , Cai Y , Zuo Q , Zhang L , Dong S , Liu Y . 2023. Chromosome‐level genome assembly of *Niphotrichum japonicum* provides new insights into heat stress responses in mosses. Frontiers in Plant Science 14: 1271357. 37920716 10.3389/fpls.2023.1271357 PMC10619864

