# Supplementary material for: CNGCs in Marchantia paleacea uncouple arbuscular mycorrhizal symbiosis and rhizoid development
Source: New Phytol. 2026 Jun 4;251(4):2027–40. doi: 10.1111/nph.71296 (PMC13373866; doi:10.1111/nph.71296)
Supplement: Supplementary file 1 — Fig. S1 Marchantia paleacea contains two co‐orthologues to the angiosperm Group I, II and III CNGCs. Fig. S2 Expression analysis of MpaCNGC promoters. Fig. S3 Generation of Mpacngc CRISPR/Cas9 knockout mutants. Fig. S4 Truncation of MpaCNGC proteins in CRISPR/Cas9 knockout mutants. Fig. S5 MpaCNGC3 and MpaCNGC4 are required for thallus development. Fig. S6 Nonbulged cells in the Mpacngc1/3/4 mutant are impaired in germinated spore exudate‐induced nuclear Ca2+ oscillations. [file NPH-251-2027-s003.zip › nph71296-sup-0003-FigureS1-S6-TableS1-S4@Supporting Information Figs S1-6 and Tables S2-4.docx]

## *New Phytologist* Supporting Information

**CNGCs in *Marchantia paleacea* uncouple arbuscular mycorrhizal symbiosis and rhizoid development**

Anson Ho Ching Lam, Aisling Cooke, Jake Richardson, Myriam Charpentier

Article acceptance date: 08 May 2026

The following Supporting Information is available for this article:

**Fig. S1** *Marchantia paleacea* contains two co-orthologues to the angiosperm Group I, II and III CNGCs

**Fig. S2** Expression analysis of *MpaCNGC* promoters

**Fig. S3** Generation of *Mpacngc* CRISPR/Cas9 knockout mutants

**Fig. S4** Truncation of MpaCNGC proteins in CRISPR/Cas9 knockout mutants.

**Fig. S5** *MpaCNGC3* and *MpaCNGC4* are required for thallus development

**Fig. S6** Non-bulged cells in the *Mpacngc1/3/4* mutant are impaired in germinated spore exudate-induced nuclear Ca^2+^ oscillations

**Table S1** List of sequence accessions used for phylogenetic analysis

**Table S2** List of primers used in this study

**Table S3** List of level 1 and level 2 Golden Gate vectors generated in this study

**Table S4** List of accession numbers for *Marchantia* genes used in this study.

**Movie S1** 3D reconstruction of a *Mpacngc3/4 #3* plant colonized by *Rhizophagus irregularis* through a bulged cell.

**Movie S2** 3D reconstruction of a *Mpacngc3/4 #3* plant colonized by *Rhizophagus irregularis* through a non-bulged cell.

**Fig. S1 *Marchantia paleacea* contains two co-orthologues to the angiosperm Group I, II and III CNGCs.** Maximum likelihood phylogeny of CNGCs in the genomes of land plants, including eleven angiosperms (Am: *Amborella trichopoda*, At: *Arabidopsis thaliana*, Bd: *Brachypodium distachyon*, Gm: *Glycine max,* Mt: *Medicago truncatula*, Os: *Oryza sativa*, Pt: *Populus trichocarpa*, Si: *Setaria italica*, Sl: *Solanum lycopersicum*, Vv: *Vitis vinifera,* Zm: *Zea mays*), one lycophyte (Sm: *Selaginella moellendorffii*), four hornworts (Aa: *Anthoceros agrestis* [Oxford], Mf: *Megaceros flagellaris*, No: *Notothylas orbicularis*, Pc: *Phaeoceros carolinianus*), five liverworts (Ae: *Apopellia endiviifolia*, Lc: *Lunularia cruciata*, Mpa: *Marchantia paleacea,* Mp: *Marchantia polymorpha*, Rs: *Riccia sorocarpa* Bisch.), five mosses (Hc: *Hypnum curvifolium*, Nj: *Niphotrichum japonicum*, Pp: *Physcomitrella patens*, Spfa: *Sphagnum fallax,* Tl: *Takakia lepidozioides*), twenty-six charophytes (Cbr: *Cylindrocystis brebissonii*, Cc: *Cylindrocystis cushleckae*, Cco: *Cosmocladium cf. constrictum*, Ci: *Coleochaete irregularis*, Co: *Cosmarium ochthodes*, Cs: *Coleochaete scutata*, Csu: *Cosmarium subtumidum*, Cyl: *Cylindrocystis sp.*, Ef: *Entransia fimbriat*, Gk: *Gonatozygon kinahanii*, Ip: *Interfilum paradoxum*, Ks: *Klebsormidium subtile*, Mb: *Mesotaenium braunii*, Mk: *Mesotaenium kramstei*, Ms: *Mougeotia sp.*, Ol: *Onychonema leave*, Pe: *Penium exiguum*, Pn: *Phymatodocis nordstedtiana*, Po: *Planotaenium ohtanii*, Ro: *Roya obtuse*, Sc: *Staurodesmus convergens*, So: *Staurodesmus omearii*, Spsp: *Spirotaenia sp.*, Ss: *Staurastrum sebaldi*, Xa: *Xanthidium antilopaeum*, Zs: *Zygnemopsis sp*.), and one chlorophyte (Cr: *Chlamydomonas reinhardtii*). The tree is rooted with CNGCs from *Chlamydomonas reinhardtii*. The amino acid sequences are available in Table S1. Blue dots on branches represent ultrafast bootstrap support over 90% (1000 iterations). The scale bar indicates substitutions per site. Internal dashed lines connect the ends of branches to their corresponding labels.


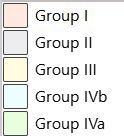


Angiosperms

Lycophytes

*Marchantia paleacea*

Bryophytes

Charophytes

Chlorophytes


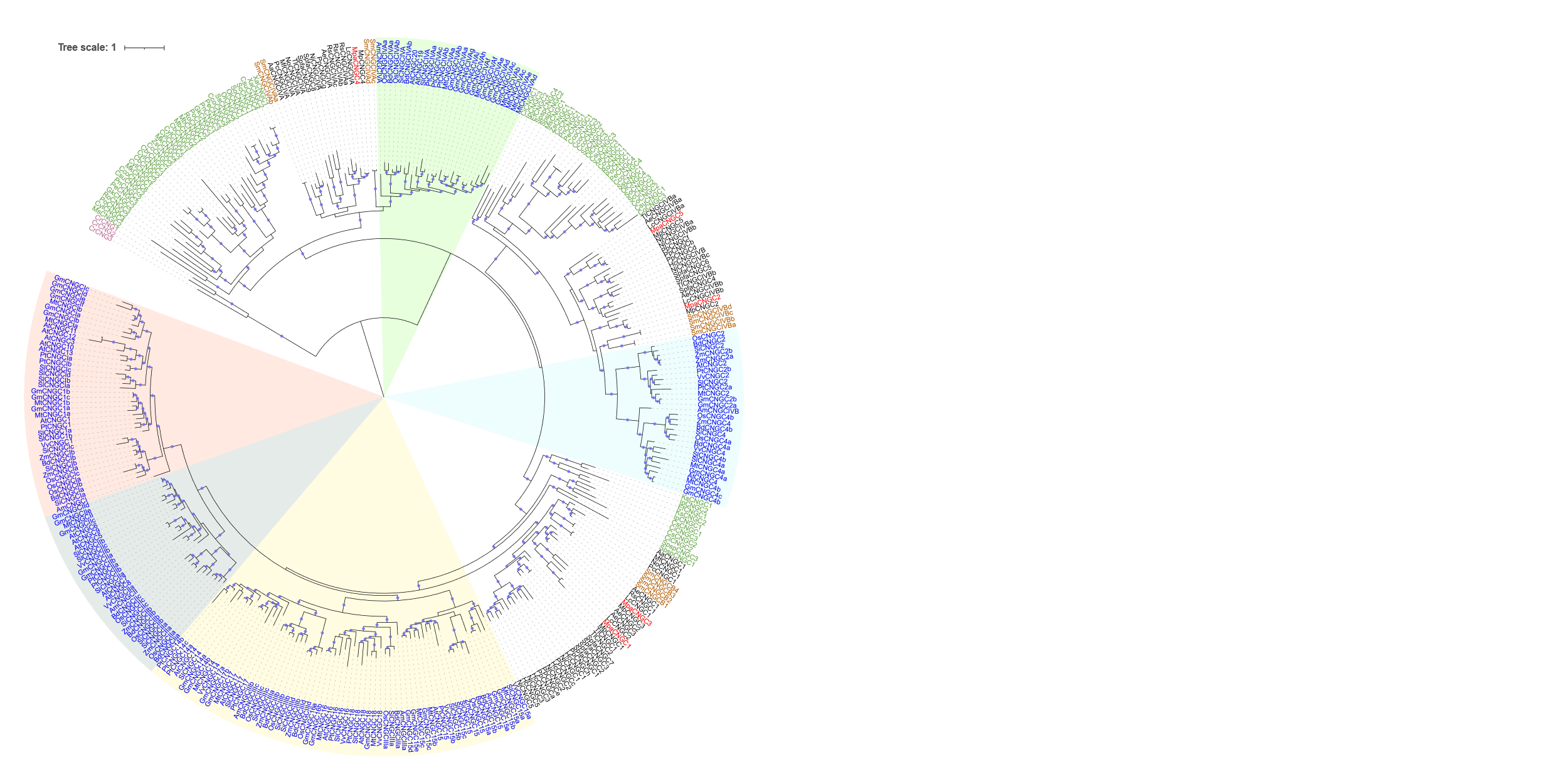


**Fig. S2 Expression analysis of *MpaCNGC* promoters.** (a-e) Representative bright-field images of two-week-old *M. paleacea* expressing *beta-glucuronidase* (*GUS*) driven by the promoters of *MpaCNGCs.* Promoter sequences of 1.5 kb, 1.5 kb, 2 kb and 1.5 kb upstream of the translation start site were used for *MpaCNGC1*, *MpaCNGC2*, *MpaCNGC3*, and *MpaCNGC4*, respectively. Scale bars represent 500 µm in (a,b,d,e), 50 µm in (c). The pictures are representative of two independent lines, with three biological replicates per line.

**
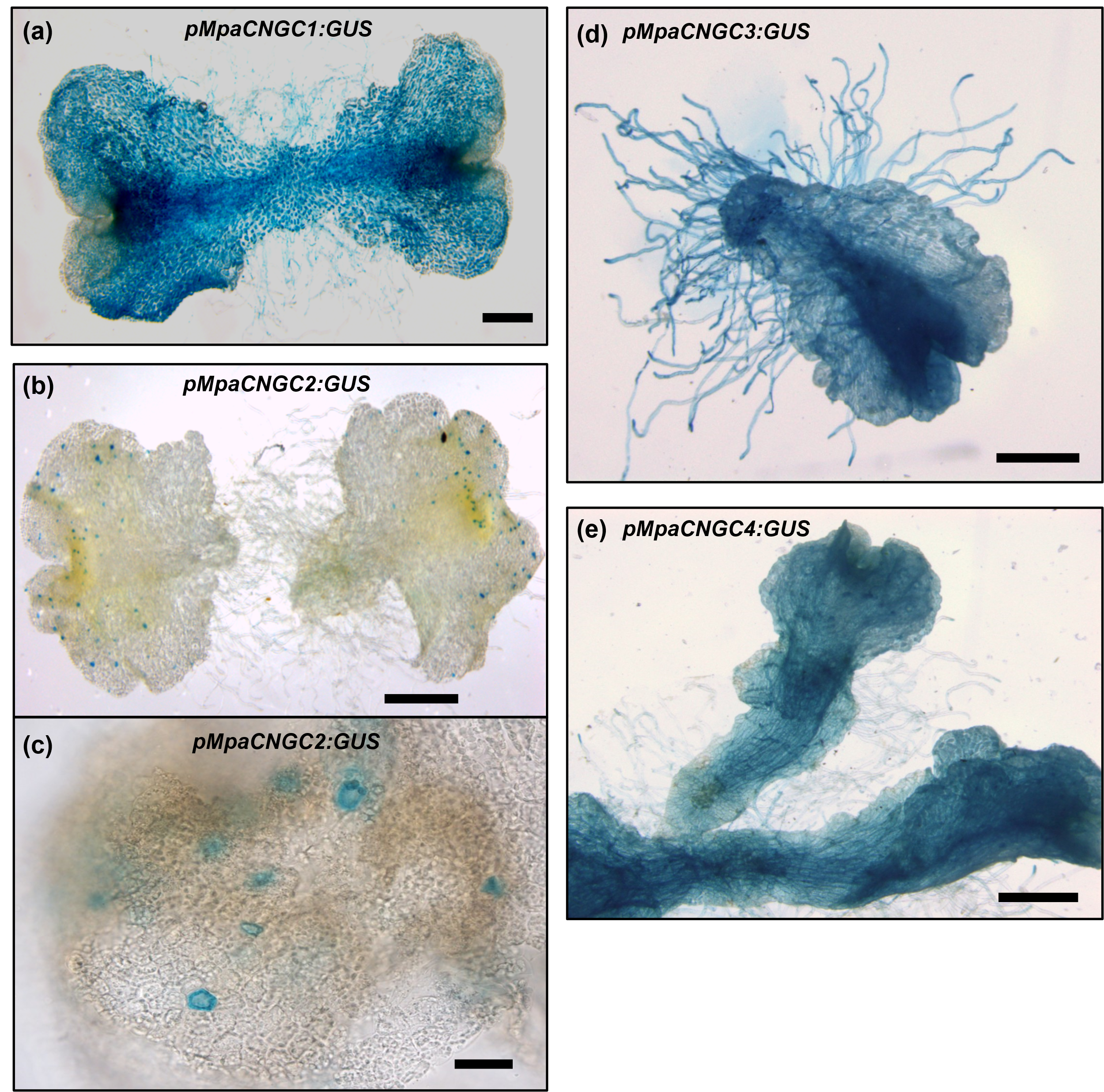
**

**Fig. S3 Generation of *Mpacngc* CRISPR/Cas9 knockout mutants.** Diagram showing the exon-intron structure of the *MpaCNGC1*, *MpaCNGC2, MpaCNGC3* and *MpaCNGC4* genomic loci. Black boxes represent exons, and lines represent introns. The positions targeted by guide RNAs (gRNAs) are indicated. Below, genomic sequences show alignments of the mutated regions at gRNA target sites. gRNA target sequences are highlighted in yellow, protospacer adjacent motif (PAM) are underlined, nucleotide substitutions are shown in red, and nucleotide deletions are indicated by red hyphens.

**
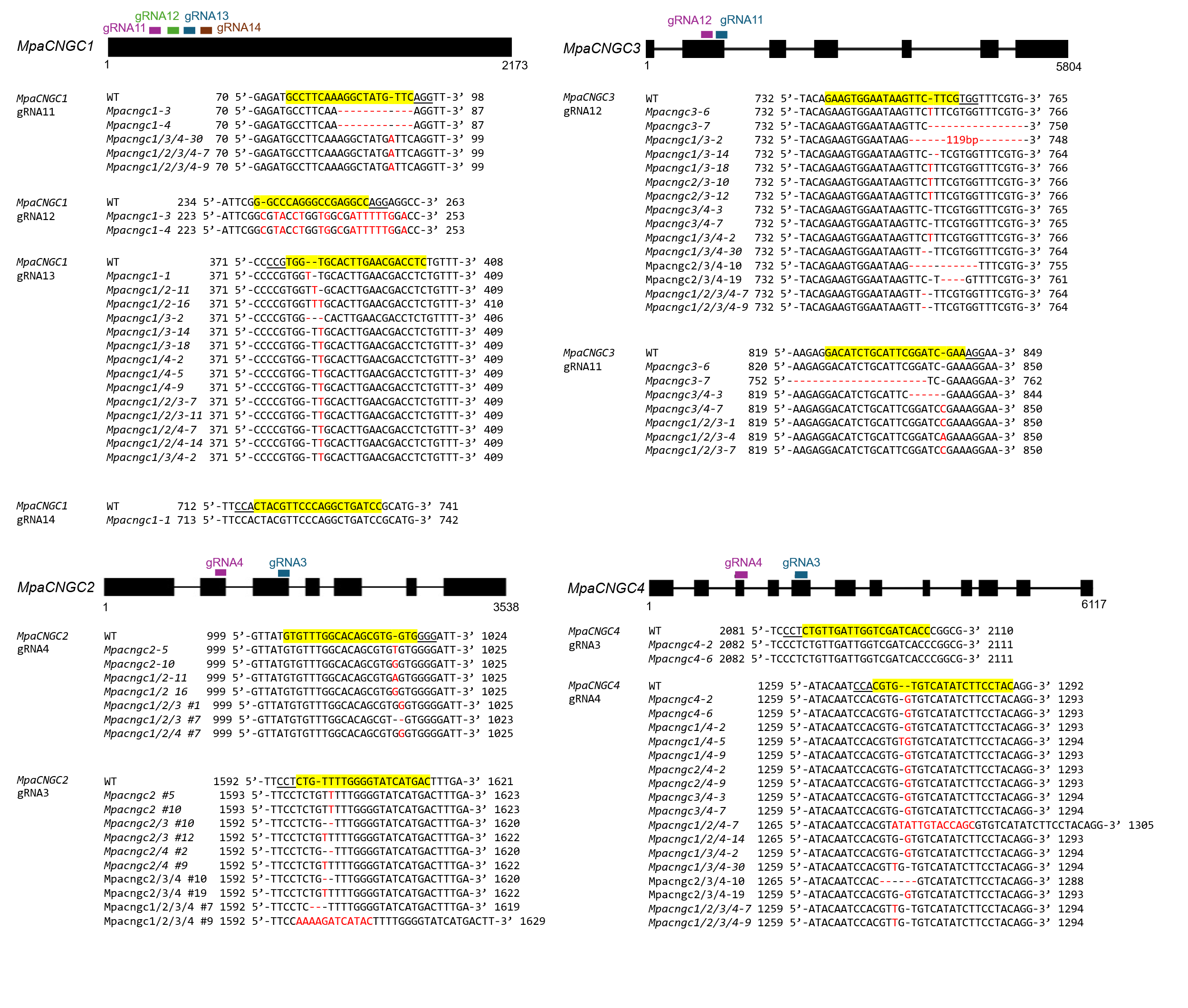
**

**Fig. S4 Truncation of MpaCNGC proteins in CRISPR/Cas9 knockout mutants.** Graphical representations of amino acid sequences showing the predicted truncated proteins in *Mpacngc* single, double, triple and quadruple mutants. Yellow boxes represent transmembrane domains, and red regions represent mistranslated amino acid sequences resulting from frame shift. Numbers at C-termini indicate protein length.

**
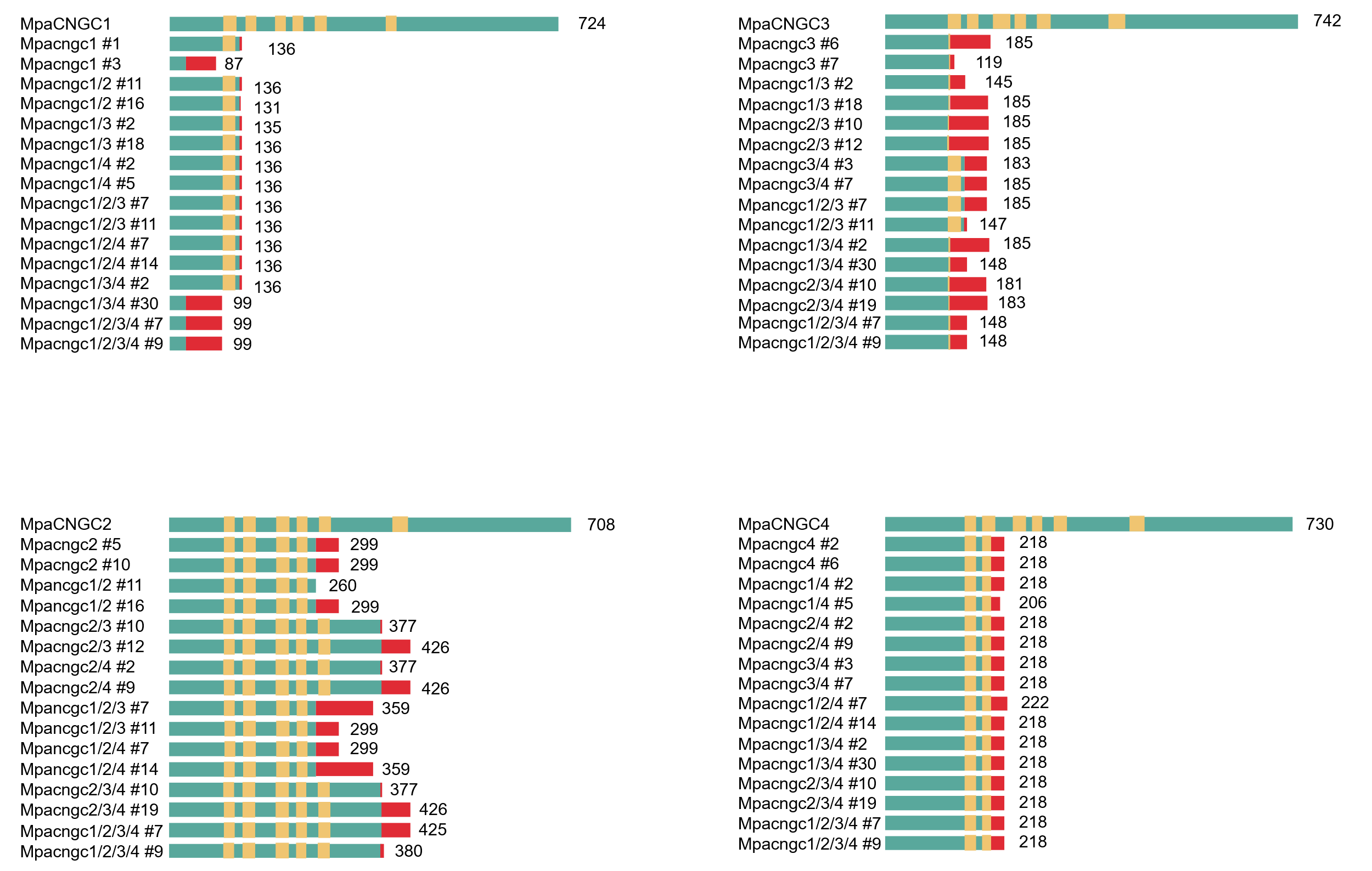
**

**Fig. S5** ***MpaCNGC3* and *MpaCNGC4* are required for thallus development.** (a) Representative pictures of four-week-old *M. paleacea* wild type expressing *Cas9* (WT*::Cas9*) and *Mpacngc* mutants (*Mpacngc3*, *Mpacngc4*, *Mpacngc3/4*, *Mpacngc1/3/4*, *Mpacngc2/3/4* and *Mpacngc1/2/3/4)* regenerated from cut thalli. Scale bar: 1 cm. (b) Scatter dot plot with bar representing thallus area of WT*::Cas9* and the indicated *Mpacngc* mutants after four weeks of growth from cut thalli. Bars and error bars indicate the mean and standard deviation, respectively. Data represent three biological replicates, with n≥46. Statistical significance was assessed using a Kruskal-Wallis test with multiple comparison to WT*::Cas9*. ^∗∗∗∗^: *p* < 0.0001.


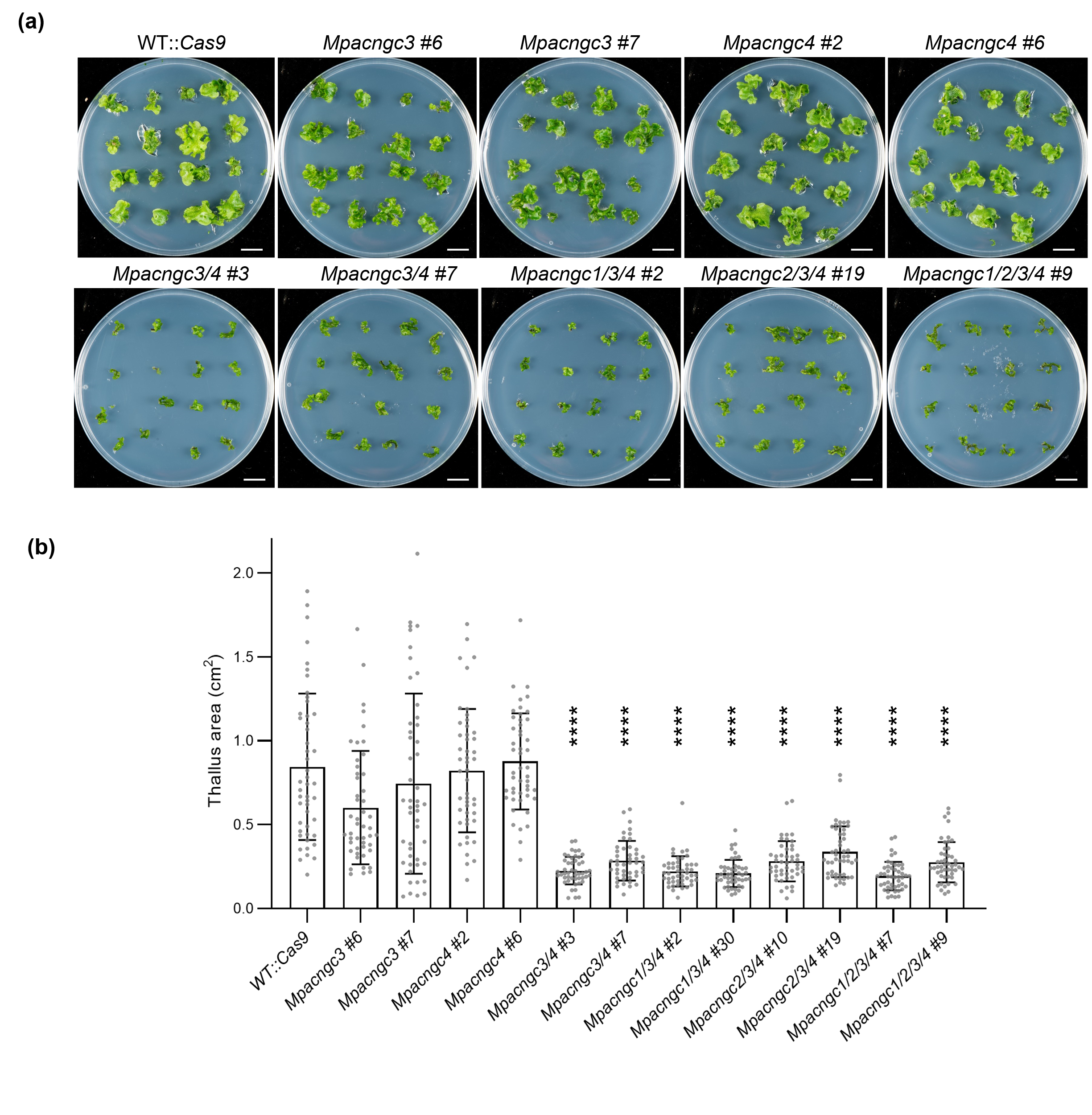


**Fig. S6 Non-bulged cells in the *Mpacngc1/3/4* mutant are impaired in germinated spore exudate-induced nuclear Ca^2+^ oscillations.** Representative *R. irregularis* germinated spore exudate (GSE)-induced calcium oscillation in non-bulge cells of *M. paleacea* *Mpacngc3/4* and *Mpacngc1/3/4* mutants. All lines express nuclear-localized *YELLOW CAMELEON 3.6* (*NLS:YC3.6*) under the control of *M. polymorpha EF1α* promoter. Traces show the ratio of yellow fluorescent protein (YFP) to cyan fluorescent protein (CFP) fluorescence in arbitrary units (A.U.). Arrowheads indicate the timing of GSE application. Numbers indicate the number of responding plants and nuclei relative to the total number analysed. Scale bar indicates 20 minutes.


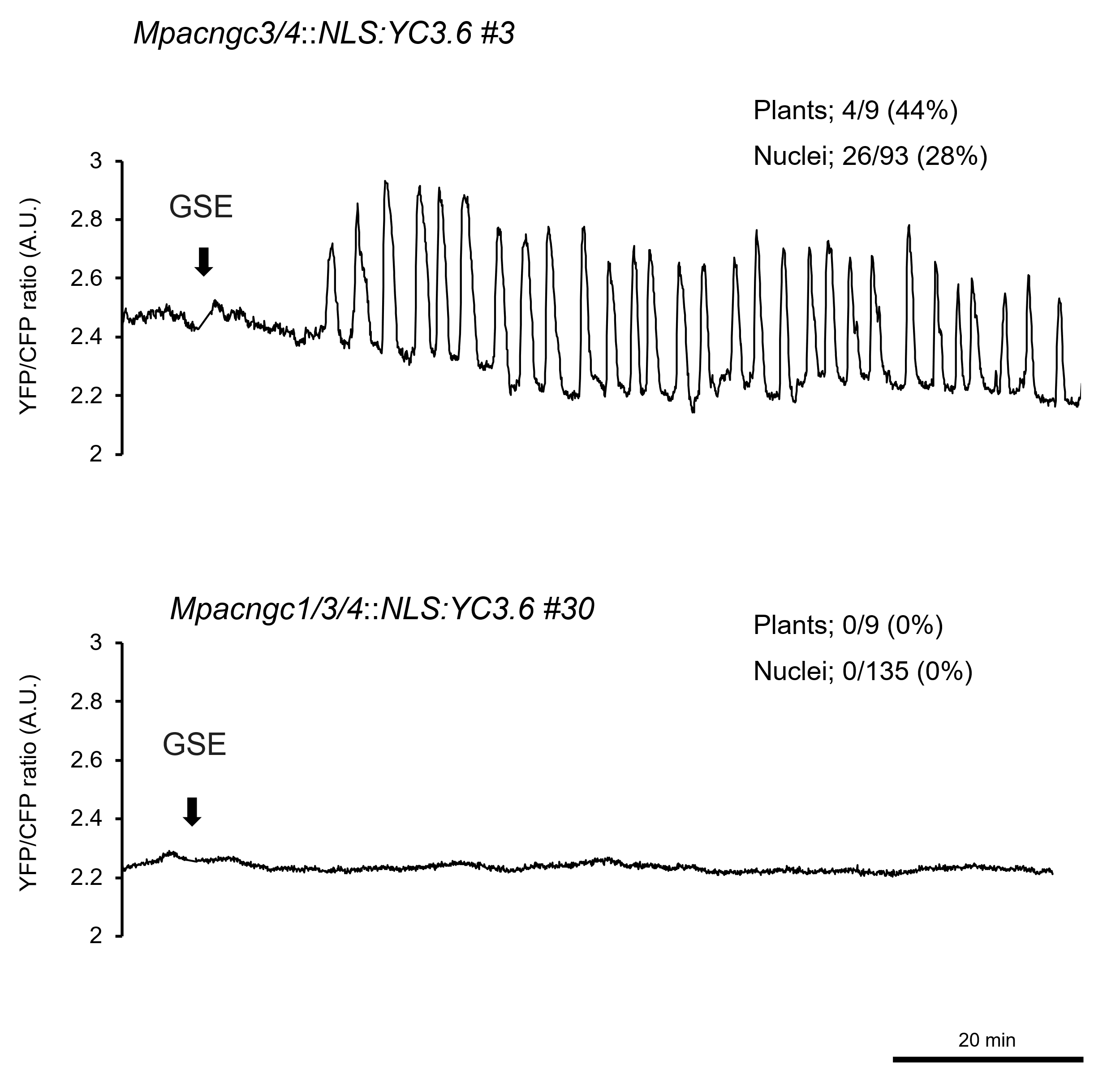
**Table S2** List of primers used in this study

| Used in figure | Oligonucleotide names | Sequence | Purpose |
| --- | --- | --- | --- |
| Fig. 4 | RiEF1a_F3 | TCTGGAGATGCCGCTATCGTTAAA | RT-qPCR for *RiEF1a* |
|  | RiEF1a_R3 | CTCTTACAGCGAAACGACCTAAAGG |  |
|  | STR_3F | TGCTCGTCTATCTCCCGTTC | RT-qPCR for *MpaSTR* |
|  | STR_3R | ACGAAGGAGTTGGTGGTGAT |  |
|  | MpaAMT2_F | AGGATCTCGCTCAGGTTGAAA | RT-qPCR for *MpaAMT2* |
|  | MpaAMT2_R | CGAGGTTGGTCATGGTGAG |  |
|  | MpaSCR_F | CAACGCAGCAACGCAAG | RT-qPCR for *MpaSCR* |
|  | MpaSCR_R | GAGGCACAGATCTTTCCATCC |  |
|  | MpaEF1a_F | GACACTACCATTCTCTTCCATTGT | RT-qPCR for *MpaEF1α* |
|  | MpaEF1a_R | CCAGGAACCACCTCATCAAAG |  |
| Fig. 1c, 4 | MpaAPT_2F | GGGTACACTTGCTGCAGGAA | RT-qPCR for *MpaAPT* |
|  | MpaAPT_2R | CTCACGGCCCTTTAGATCCG |  |
| Fig. 2 | MpaCNGC1gRNA11F | CACTCTGTGGTCTCATCTCGCCTTCAAAGGCTATGTTCGTTTTGAGACCACGAAGTG | construction of plasmid for CRISPR/Cas9 targeting *MpaCNGC1* |
|  | MpaCNGC1gRNA11R | CACTTCGTGGTCTCAAAACGAACATAGCCTTTGAAGGCGAGATGAGACCACAGAGTG |  |
|  | MpaCNGC1gRNA12F | CACTCTGTGGTCTCATCTCGGCCCAGGGCCGAGGCCAGGGTTTTGAGACCACGAAGTG |  |
|  | MpaCNGC1gRNA12R | CACTTCGTGGTCTCAAAACCCTGGCCTCGGCCCTGGGCCGAGATGAGACCACAGAGTG |  |
|  | MpaCNGC1sgRNA13F | GTGGTCTCATCTCGAGGTCGTTCAAGTGCACCAGTTTTGAGACCAC |  |
|  | MpaCNGC1sgRNA13R | GTGGTCTCAAAACTGGTGCACTTGAACGACCTCGAGATGAGACCAC |  |
|  | MpaCNGC1sgRNA14F | GTGGTCTCATCTCGGATCAGCCTGGGAACGTAGGTTTTGAGACCAC |  |
|  | MpaCNGC1sgRNA14R | GTGGTCTCAAAACCTACGTTCCCAGGCTGATCCGAGATGAGACCAC |  |
|  | MpaCNGC2sgRNA3_FWD | cactctgtggtctcatctcGTCATGATACCCCAAAACAGgttttgagaccacgaagtg | construction of plasmid for CRISPR/Cas9 targeting *MpaCNGC2* |
|  | MpaCNGC2sgRNA3_REV | CACTTCGTGGTCTCAAAACCTGTTTTGGGGTATCATGACGAGATGAGACCACAGAGTG |  |
|  | MpaCNGC2sgRNA4_FWD | cactctgtggtctcatctcGTGTTTGGCACAGCGTGGTGgttttgagaccacgaagtg |  |
|  | MpaCNGC2sgRNA4_REV | CACTTCGTGGTCTCAAAACCACCACGCTGTGCCAAACACGAGATGAGACCACAGAGTG |  |
|  | MpaCNGC3_gRNA11F | CACTCTGTGGTCTCATCTCGACATCTGCATTCGGATCGAAGTTTTGAGACCACGAAGTG | construction of plasmid for CRISPR/Cas9 targeting *MpaCNGC3* |
|  | MpaCNGC3_gRNA11R | CACTTCGTGGTCTCAAAACTTCGATCCGAATGCAGATGTCGAGATGAGACCACAGAGTG |  |
|  | MpaCNGC3_gRNA12F | CACTCTGTGGTCTCATCTCGAAGTGGAATAAGTTCTTCGGTTTTGAGACCACGAAGTG |  |
|  | MpaCNGC3_gRNA12R | CACTTCGTGGTCTCAAAACCGAAGAACTTATTCCACTTCGAGATGAGACCACAGAGTG |  |
|  | MpaCNGC4sgRNA3_FWD | cactctgtggtctcatctcGGTGATCGACCAATCAACAGgttttgagaccacgaagtg | construction of plasmid for CRISPR/Cas9 targeting *MpaCNGC4* |
|  | MpaCNGC4sgRNA3_REV | CACTTCGTGGTCTCAAAACCTGTTGATTGGTCGATCACCGAGATGAGACCACAGAGTG |  |
|  | MpaCNGC4sgRNA4_FWD | cactctgtggtctcatctcGTAGGAAGATATGACACACGgttttgagaccacgaagtg |  |
|  | MpaCNGC4sgRNA4_REV | CACTTCGTGGTCTCAAAACCGTGTGTCATATCTTCCTACGAGATGAGACCACAGAGTG |  |
|  | CNGC1_genotype_fwd | GATCATCAGTGGTGGTTGGG | Genotyping *Mpacngc1* |
|  | CNGC1_genotype_rev | ATCACCGTTCGGAGGACCGT |  |
|  | MpaCNGC1_F2 | CTCCAGGAGCCTCGTCTTTG |  |
|  | MpaCNGC1_R2 | CCAGACCGTCTCCATAAATACTC |  |
|  | CNGC1_3R | AAATACTCCCGTGTCCTGGG |  |
|  | CNGC2_genotype_F | CCAGTACATACCAAAGGTCTTGC | Genotyping *Mpacngc2* |
|  | CNGC2_genotype_R | GGTATGCAGCTTCTTAAACATTGTATAATAG |  |
|  | Genotype_CNGC3KO_fwd2 | CAGATAAAGGTTCAGAGGCC | Genotyping *Mpacngc3* |
|  | CNGC3_genotype_rev | AGAGGTAGTACAGCCACCAA |  |
|  | MpaCNGC3_F2 | TGTTATCGAACTAGTGCTGTCTG |  |
|  | MpaCNGC3_R2 | ACTAATTCCGCAGGTGGATAAG |  |
|  | CNGC4_genotype_F3 | GTCTTCAACAATGCCGCTGC | Genotyping *Mpacngc4* |
|  | CNGC4_genotype_R4 | TACATGAGCAGATTGATGACAAAG |  |
|  | CNGC4_genotype_F_new | GGACTACAAGTGCTTGGTCTTC |  |
|  | CNGC4_genotype_R_new | CTACATGACCAGCAAGCAGG |  |
| Fig. 1b | CNGC1_N_term_F | ggggacaagtttgtacaaaaaagcaggcttcATGAAGGAGGCATTTACTTATC | RT-PCR for *MpaCNGC1* |
|  | CNGC1_C_term_R | ggggaccactttgtacaagaaagctgggtcCTATATGTGGCTGTCATCTC |  |
|  | CNGC2_N_term_F | ggggacaagtttgtacaaaaaagcaggcttcATGAAGATGGGGCGGAAAG | RT-PCR for *MpaCNGC2* |
|  | CNGC2_C_term_R | ggggaccactttgtacaagaaagctgggtcTCACTCCAAATGATCTTGAG |  |
|  | CNGC3_N_term_F | ggggacaagtttgtacaaaaaagcaggcttcATGAATCATGGGCATGGG | RT-PCR for *MpaCNGC3* |
|  | CNGC3_C_term_R | ggggaccactttgtacaagaaagctgggtcCTAATCATCTTCCACGGTG |  |
|  | CNGC4_N_term_F | ggggacaagtttgtacaaaaaagcaggcttcATGGGCAGGAATGGACG | RT-PCR for *MpaCNGC4* |
|  | CNGC4_C_term_R | ggggaccactttgtacaagaaagctgggtcCTACTTGGACCTGTCAGAC |  |
|  | CNGC5_N_term_F | ggggacaagtttgtacaaaaaagcaggcttcATGCCTTTGGCCATCAGTTC | RT-PCR for *MpaCNGC5* |
|  | CNGC5_C_term_R | ggggaccactttgtacaagaaagctgggtcCTACTCAAGATGAGTCGAAGGTTTTG |  |
| Fig. 1c | CNGC1_2F | GCTACTCACTTGGGCTTTGG | RT-qPCR for *MpaCNGC1* |
|  | CNGC1_2R | AGAGAGAAAAGGCCTCCACC |  |
|  | MpaCNG2-qPCR-1534F | ATTTGCGAACGGTTGAAGCC | RT-qPCR for *MpaCNGC2* |
|  | MpaCNG2-qPCR-1725R | ACACCACGACAACAGTTCGT |  |
|  | CNGC3_2F | CGTGGTCATCTGGAGAGTGT | RT-qPCR for *MpaCNGC3* |
|  | CNGC3_2R | ACGCCCAAGTAAGAAGCTCT |  |
|  | MpaCNG4-qPCR-1439F | TTTGGATGAAGCGACGTCGA | RT-qPCR for *MpaCNGC4* |
|  | MpaCNG4-qPCR-1512R | AACCCATCTGAATCGCCCAG |  |
|  | CNGC5_qPCR_F | GCCAGGTGCCACTTTTCG | RT-qPCR for *MpaCNGC5* |
|  | CNGC5_qPCR_R | CTTAGCACCATCTCACCCTTG |  |

**Table S3** List of level 1 and level 2 Golden Gate vectors used in this study

| L1 construct name | Backbone | Promoter | CDS | C-term tag | Terminator |
| --- | --- | --- | --- | --- | --- |
| AL15030 | R1 pICH47802 | AL15058 p35S short | AL15069 hptII |  | pICH41421 tNOS |
| L1AC347 | R2 pICH47811 | AL10785 pMpEF1a | AL25022 NLS-YC3.6 |  | pICH41421 tNOS |
| L1-R3-19 | R3 pICH47822 | AL15058 p35S short | AL15071 mCherry |  | pICH41421 tNOS |
| L1AC186 | R2 pICH47811 | AL10785 pMpEF1a | AL15747 Cas9 codon optimized for *A. thaliana* |  | pICH41421 tNOS |
| L1AC365 | R3 pICH47822 | AL10785 pMpEF1a | AL25022 NLS-YC3.6 |  | pICH41414 t35S |
| L1AC348 | R5 pICH47841 | AL10785 pMpEF1a | AL25022 NLS-YC3.6 |  | pICH41421 tNOS |
| AL1080 | R1 pICH47802 | AL15058 p35S short | mALS domesticated |  | pICH41421 tNOS |
| L1AC366 | R3 AL32983 pMpaU6-1 |  | Mpacngc1 gRNA11 |  |  |
| L1AC367 | R4 AL32984 pMpaU6-1 |  | Mpacngc1 gRNA12 |  |  |
| L1AC335 | R3 AL32983 pMpaU6-1 |  | Mpacngc1 gRNA13 |  |  |
| L1AC336 | R4 AL32984 pMpaU6-1 |  | Mpacngc1 gRNA14 |  |  |
| L1AC339 | R3 AL32983 pMpaU6-1 |  | Mpacngc2 gRNA3 |  |  |
| L1AC340 | R4 AL32984 pMpaU6-1 |  | Mpacngc2 gRNA4 |  |  |
| L1AC368 | R3 AL32983 pMpaU6-1 |  | Mpacngc3 gRNA11 |  |  |
| L1AC369 | R4 AL32984 pMpaU6-1 |  | Mpacngc3 gRNA12 |  |  |
| L1AC343 | R3 AL32983 pMpaU6-1 |  | Mpacngc4 gRNA3 |  |  |
| L1AC344 | R4 AL32984 pMpaU6-1 |  | Mpacngc4 gRNA4 |  |  |
| AL1028 | R5 AL1025 pMpaU6-1 |  | Mpacngc4 gRNA4 |  |  |
| AL1087 | R5 AL1025 pMpaU6-1 |  | Mpacngc3 gRNA11 |  |  |
| AL1023 | R6 plCH47852 | AL10785 pMpEF1a | AL25022 NLS-YC3.6 |  | pICH41421 tNOS |
| L1AC219 | R2 pICH47811 | EC52414 pMpaCNGC1 | AL75111 GUS |  | pICH41414 t35S |
| L1AC220 | R2 pICH47811 | EC52415 pMpaCNGC2 | AL75111 GUS |  | pICH41414 t35S |
| L1AC221 | R2 pICH47811 | EC52416 pMpaCNGC3 | AL75111 GUS |  | pICH41414 t35S |
| L1AC222 | R2 pICH47811 | EC52417 pMpaCNGC4 | AL75111 GUS |  | pICH41414 t35S |

| L2 construct name | Backbone | Position 1 | Position 2 | Position 3 | Position 4 | Position 5 | Position 6 | Linker |
| --- | --- | --- | --- | --- | --- | --- | --- | --- |
| L2AC381 | 50507 | AL15030 p35S:hptII | L1AC186 pMpEF1a:Cas9 | L1AC365 pMpEF1a:NLS-YC3.6 |  |  |  | pICH41766 |
| AC367 | 50507 | AL15030 p35S:hptII | L1AC186 pMpEF1a:Cas9 | L1AC366 Mpacngc1 gRNA11 | L1AC367 Mpacngc1 gRNA12 | L1AC348 pMpEF1a:NLS-YC3.6 |  | pICH41800 |
| AC365 | 50507 | AL15030 p35S:hptII | L1AC186 pMpEF1a:Cas9 | L1AC335 Mpacngc1 gRNA13 | L1AC336 Mpacngc1 gRNA14 | L1AC348 pMpEF1a:NLS-YC3.6 |  | pICH41800 |
| AL2018 | 50507 | AL15030 p35S:hptII | L1AC186 pMpEF1a:Cas9 | L1AC339 Mpacngc2 gRNA3 | L1AC340 Mpacngc2 gRNA4 | L1AC348 pMpEF1a:NLS-YC3.6 |  | pICH41800 |
| AC369 | 50507 | AL15030 p35S:hptII | L1AC186 pMpEF1a:Cas9 | L1AC368 Mpacngc3 gRNA11 | L1AC369 Mpacngc3 gRNA12 | L1AC348 pMpEF1a:NLS-YC3.6 |  | pICH41800 |
| AL2022 | 50507 | AL15030 p35S:hptII | L1AC186 pMpEF1a:Cas9 | L1AC343 Mpacngc4 gRNA3 | L1AC344 Mpacngc4 gRNA4 | L1AC348 pMpEF1a:NLS-YC3.6 |  | pICH41800 |
| AL2123 | 50507 | AL15030 p35S:hptII | L1AC186 pMpEF1a:Cas9 | L1AC335 Mpacngc1 gRNA13 | L1AC340 Mpacngc2 gRNA4 | L1AC348 pMpEF1a:NLS-YC3.6 |  | pICH41800 |
| AL2017 | 50507 | AL15030 p35S:hptII | L1AC186 pMpEF1a:Cas9 | L1AC335 Mpacngc1 gRNA13 | L1AC369 Mpacngc3 gRNA12 | L1AC348 pMpEF1a:NLS-YC3.6 |  | pICH41800 |
| AL2099 | 50507 | AL15030 p35S:hptII | L1AC186 pMpEF1a:Cas9 | L1AC335 Mpacngc1 gRNA13 | L1AC344 Mpacngc4 gRNA4 | L1AC348 pMpEF1a:NLS-YC3.6 |  | pICH41800 |
| AL2107 | 50507 | AL15030 p35S:hptII | L1AC186 pMpEF1a:Cas9 | L1AC339 Mpacngc2 gRNA3 | L1AC369 Mpacngc3 gRNA12 | L1AC348 pMpEF1a:NLS-YC3.6 |  | pICH41800 |
| AL2108 | 50507 | AL15030 p35S:hptII | L1AC186 pMpEF1a:Cas9 | L1AC339 Mpacngc2 gRNA3 | L1AC344 Mpacngc4 gRNA4 | L1AC348 pMpEF1a:NLS-YC3.6 |  | pICH41800 |
| AL2033 | 50507 | AL15030 p35S:hptII | L1AC186 pMpEF1a:Cas9 | L1AC368 Mpacngc3 gRNA11 | L1AC369 Mpacngc3 gRNA12 | AL1028 Mpacngc4 gRNA4 | AL1023 pMpEF1a:NLS-YC3.6 | pICH41822 |
| AL2124 | 50507 | AL15030 p35S:hptII | L1AC186 pMpEF1a:Cas9 | L1AC335 Mpacngc1 gRNA13 | L1AC340 Mpacngc2 gRNA4 | AL1087 Mpacngc3 gRNA11 | AL1023 pMpEF1a:NLS-YC3.6 | pICH41822 |
| AL2125 | 50507 | AL15030 p35S:hptII | L1AC186 pMpEF1a:Cas9 | L1AC335 Mpacngc1 gRNA13 | L1AC340 Mpacngc2 gRNA4 | AL1028 Mpacngc4 gRNA4 | AL1023 pMpEF1a:NLS-YC3.6 | pICH41822 |
| AL2024 | 50507 | AL15030 p35S:hptII | L1AC186 pMpEF1a:Cas9 | L1AC366 Mpacngc1 gRNA11 | L1AC369 Mpacngc3 gRNA12 | AL1028 Mpacngc4 gRNA4 | AL1023 pMpEF1a:NLS-YC3.6 | pICH41822 |
| AL2100 | 50507 | AL15030 p35S:hptII | L1AC186 pMpEF1a:Cas9 | L1AC335 Mpacngc1 gRNA13 | L1AC369 Mpacngc3 gRNA12 | AL1028 Mpacngc4 gRNA4 | AL1023 pMpEF1a:NLS-YC3.6 | pICH41822 |
| AL2109 | 50507 | AL15030 p35S:hptII | L1AC186 pMpEF1a:Cas9 | L1AC339 Mpacngc2 gRNA3 | L1AC369 Mpacngc3 gRNA12 | AL1028 Mpacngc4 gRNA4 | AL1023 pMpEF1a:NLS-YC3.6 | pICH41822 |
| AL2110 | 50507 | AL1080 p35S:mALS | L1AC186 pMpEF1a:Cas9 | L1AC339 Mpacngc2 gRNA3 |  |  |  | pICH41766 |
| L2AC219 | 50507 | AL15030 p35S:hptII | L1AC219 pMpaCNGC1:GUS | L1-R3-19 p35S:mCherry |  |  |  | pICH41766 |
| L2AC220 | 50507 | AL15030 p35S:hptII | L1AC220 pMpaCNGC2:GUS | L1-R3-19 p35S:mCherry |  |  |  | pICH41766 |
| L2AC221 | 50507 | AL15030 p35S:hptII | L1AC221  pMpaCNGC3:GUS | L1-R3-19 p35S:mCherry |  |  |  | pICH41766 |
| L2AC222 | 50507 | AL15030 p35S:hptII | L1AC222 pMpaCNGC4:GUS | L1-R3-19 p35S:mCherry |  |  |  | pICH41766 |

p: promoter; t: terminator; 35S: 35S Cauliflower Mosaic Virus (CaMV); NLS-YC3.6: nuclear-localized signal fused to Ca^2+^ reporter *Yellow Cameleon version 3.6* ; EF1a: *ELONGATION FACTOR 1α;* mALS: *ACETOLACTATE SYNTHASE*; NOS: *NOPALINE SYNTHASE*; hptII: *HYGROMYCIN PHOSPHOTRANSFERASE*; pMpaU6-1: promoter of *M. paleacea* U6 snRNA; GUS: *β-GLUCURONIDASE*

**Table S4** List of accession numbers for *Marchantia* genes used in this study.

| Gene | Species | Accession |
| --- | --- | --- |
| *MpCNGC1* | *Marchantia polymorpha* | Mp4g04110 |
| *MpCNGC2* | *Marchantia polymorpha* | Mp3g14660 |
| *MpCNGC3* | *Marchantia polymorpha* | Mp6g01920 |
| *MpCNGC4* | *Marchantia polymorpha* | Mp5g07780 |
| *MpCNGC5* | *Marchantia polymorpha* | Mp4g11640 |
| *MpaCNGC1* | *Marchantia paleacea* | Marpal_utg000008g0018121 |
| *MpaCNGC2* | *Marchantia paleacea* | Marpal_utg000163g0184561 |
| *MpaCNGC3* | *Marchantia paleacea* | Marpal_utg000029g0062841 |
| *MpaCNGC4* | *Marchantia paleacea* | Marpal_utg000020g0054311 |
| *MpaCNGC5* | *Marchantia paleacea* | Marpal_utg000010g0021101 |
| *MpaSTR* | *Marchantia paleacea* | Marpal_utg000139g0174371 |
| *MpaAPT* | *Marchantia paleacea* | Marpal_utg000039g0074951 |
| *MpaAMT2* | *Marchantia paleacea* | Marpal_utg000013g0032291 |
| *MpaSCR* | *Marchantia paleacea* | Marpal_utg000081g0134291 |
| *MpaEF1α* | *Marchantia paleacea* | Marpal_utg000003g0004561 |
| *RiEF1a* | *Rhizophagus irregularis* | XM_025321412.1 |
| *MpEF1a* | *Marchantia polymorpha* | Mp3g23400 |
| *MpU6* | *Marchantia paleacea* | Marpal_utg000010g0020161 |

**Movie S1** 3D reconstruction of a *Mpacngc3/4 #3* plant colonized by *Rhizophagus irregularis* through a bulged cell. The cyan channel shows the cell wall stain SR2200, and the yellow channel shows the AM fungi labelled with WGA-Alexa Fluor 488. Scale bar: 20 µm. Related to Fig. 3b.

**Movie S2** 3D reconstruction of a *Mpacngc3/4 #3* plant colonized by *Rhizophagus irregularis* through a non-bulged cell. The cyan channel shows the cell wall stain SR2200, and the yellow channel shows the AM fungi labelled with WGA-Alexa Fluor 488. Scale bar: 20 µm. Related to Fig. 3b.
